# Magnesium ion hydrogel enhances resistance to radiation-induced bone injury by modulating the bone immune microenvironment and promoting microvascularization

**DOI:** 10.1093/rb/rbaf118

**Published:** 2025-12-03

**Authors:** Qiong Wang, Xinpeng Hu, Zeyu Xiao, Kunlin Ye, Jia Li, Jiaxin Tan, Nuonuo Rao, Dong Zhang, Guodong Sun, Mingxiang Cai, Ni Shao, Nianlan Cheng, Le Bai, Xiangning Liu, Changzheng Shi, Liangping Luo

**Affiliations:** Medical Imaging Center, The First Affiliated Hospital of Jinan University, Guangzhou 510630, China; Hospital of Stomatology, The First Affiliated Hospital of Jinan University, School of Stomatology, Jinan University, Guangzhou 510630, China; The Affiliated Dongguan Songshan Lake Central Hospital, Guangdong Medical University, Dongguan 523326, China; Medical Imaging Center, The First Affiliated Hospital of Jinan University, Guangzhou 510630, China; Medical Imaging Center, The First Affiliated Hospital of Jinan University, Guangzhou 510630, China; Hospital of Stomatology, The First Affiliated Hospital of Jinan University, School of Stomatology, Jinan University, Guangzhou 510630, China; Hospital of Stomatology, The First Affiliated Hospital of Jinan University, School of Stomatology, Jinan University, Guangzhou 510630, China; Hospital of Stomatology, The First Affiliated Hospital of Jinan University, School of Stomatology, Jinan University, Guangzhou 510630, China; Medical Imaging Center, The First Affiliated Hospital of Jinan University, Guangzhou 510630, China; The Fifth Affiliated Hospital (Heyuan Shenhe People’s Hospital), Jinan University, Heyuan 517000, China; Hospital of Stomatology, The First Affiliated Hospital of Jinan University, School of Stomatology, Jinan University, Guangzhou 510630, China; Medical Imaging Center, The First Affiliated Hospital of Jinan University, Guangzhou 510630, China; Medical Imaging Center, The First Affiliated Hospital of Jinan University, Guangzhou 510630, China; Medical Imaging Center, The First Affiliated Hospital of Jinan University, Guangzhou 510630, China; Hospital of Stomatology, The First Affiliated Hospital of Jinan University, School of Stomatology, Jinan University, Guangzhou 510630, China; Medical Imaging Center, The First Affiliated Hospital of Jinan University, Guangzhou 510630, China; Medical Imaging Center, The First Affiliated Hospital of Jinan University, Guangzhou 510630, China; The Fifth Affiliated Hospital (Heyuan Shenhe People’s Hospital), Jinan University, Heyuan 517000, China

**Keywords:** radiation-induced bone injury, osteoimmunity, magnesium ions, microvascularization, iron metabolism

## Abstract

Mandibular radiation-induced bone injury (RIBI) is a common, severe complication of radiotherapy with no effective treatment. The early course is clinically subtle yet pathologically complex: ionizing radiation (IR) rapidly induces microvascular dysfunction, amplifies immune-mediated inflammation and disrupts bone homeostasis. This complexity, together with safety considerations, hampers therapeutic translation. Magnesium (Mg^2+^) is an essential bone component whose pro-osteogenic activity is well established; nevertheless, irradiation may remodel the multi-target effects of bioactive ions, and the integrated mechanisms of Mg^2+^ in bone radiation injury remain to be clarified. Here, we compared local delivery of an Mg^2+^- crosslinked alginate hydrogel (Mg@Alg) under irradiated versus non-irradiated conditions in rats and combined macrophage and endothelial cell models to evaluate radioprotective effects and mechanisms. In our study, Mg@Alg attenuated bone loss and apoptosis within 14 days after IR, promoted M2-like macrophage polarization, and improved microvascular density and maturation, thereby contributing to inflammatory microenvironment remodeling. Mechanistically, Mg^2+^ intervention was accompanied by decreased ferritin, downregulation of prolyl hydroxylase domain-2 (PHD2), and stabilization of hypoxia-inducible factor-1α (HIF-1α), together with vascular endothelial growth factor A upregulation; these changes were partly reversed by Fe^2+^, suggesting an iron-dependent, PHD2/HIF-1α-biased modulation that coordinates immune homeostasis and vascular regeneration to improve immune–vascular coupling. Notably, while Mg^2+^ efficacy appeared enhanced under IR, the effective concentration window narrowed. In sum, peri-radiotherapy, localized, short-term Mg^2+^ delivery may improve bone tolerance to radiation and mitigate early RIBI. These findings provide an experimental basis for low-risk, clinically translatable bone radioprotective strategies and expand the application paradigm of magnesium-based materials in radiotherapy protection contexts.

## Graphical abstract

**Figure:**
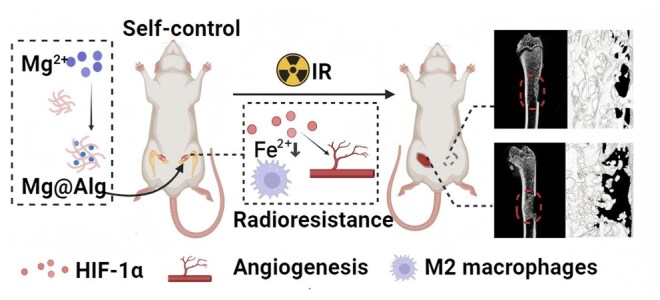

## Introduction

Approximately 50% of cancer patients undergo radiotherapy during their treatment [1, 2]. Bone tissue, characterized by low oxygen tension and limited microvascular perfusion, absorbs around 30–40% more radiation than surrounding tissues [3]. This makes it more susceptible to imbalances in bone homeostasis, leading to radiation-induced bone injury (RIBI), such as osteomyelitis, osteoporosis and osteoradionecrosis, caused by ionizing radiation (IR) [4–6]. Osteoradionecrosis of the jaw, affecting the facial region, severely imposes functional and psychosocial burdens.

RIBI pathogenesis follows a cascade amplification process [4, 7, 8]: early microvascular impairment contributes to ischemia and hypoxia in bone areas, exacerbating cellular oxidative stress and DNA damage; subsequent infiltration of inflammatory immune cells establishes a pro-inflammatory milieu, in which cytokines potentiate osteoclast activation, leading to progressive bone loss. This “hypoxia–inflammation–bone resorption” vicious cycle underlies the difficulty of achieving durable healing. Current management is largely supportive and symptom-directed—primarily surgical intervention and hyperbaric oxygen therapy—with no specific, effective pharmacotherapies available [8–10]. Pre-radiotherapy prevention relies on rigorous dental evaluation, including extraction of teeth within high-dose fields that are non-restorable or actively infected [11, 12]. However, such procedures can introduce new osseous trauma during the peri-radiotherapy period and may heighten the risk of RIBI [13].

Given the complexity of multicellular interactions and network regulation in the irradiated inflammatory bone microenvironment, an ideal intervention should emphasize prevention and coordinated, multi-target modulation. Small-molecule agents (e.g. the aminopropyl carbazole P7C3) and nanozyme-based approaches have been explored to mitigate IR-induced inflammatory injury [14, 15]. However, concerns about long-term safety, stability *in vivo*, and tissue targeting still limit clinical translation [16]. By contrast, bioactive ions offer multi-target microenvironmental regulation with good biocompatibility and have shown promise in tissue regeneration [15, 17, 18]. Magnesium ions (Mg^2+^) are essential for bone metabolism and serve as cofactors in more than 500 enzymatic reactions [19]. Mg^2+^ deficiency reduces hypoxia tolerance, elevates inflammatory cytokines and acute-phase proteins, and promotes excess free-radical formation [20–22]. Studies indicate that Mg^2+^ can dampen M1 macrophage polarization, suppress TNF-α and IL-6, and act directly or indirectly on mesenchymal stem cells and osteoblasts to accelerate osteogenesis [23–25]. Owing to their biocompatibility, bioactivity, mechanical performance and biodegradability, magnesium-based materials are considered promising for orthopedic and dental applications [26, 27].

Nevertheless, the pathological microenvironment can change the effects of multi-target therapies, with implications for both safety and sites of action [28–30]. Radiation can disturb divalent-ion homeostasis by altering membrane permeability and transporter function [31, 32]. In particular, IR increases cellular iron storage and can induce iron-dependent cell death (ferroptosis), thereby accelerating bone loss [32, 33]. These observations highlight the need to re-evaluate the overall effects and mechanisms of Mg^2+^ under radiation-specific conditions.

**Figure 1.**
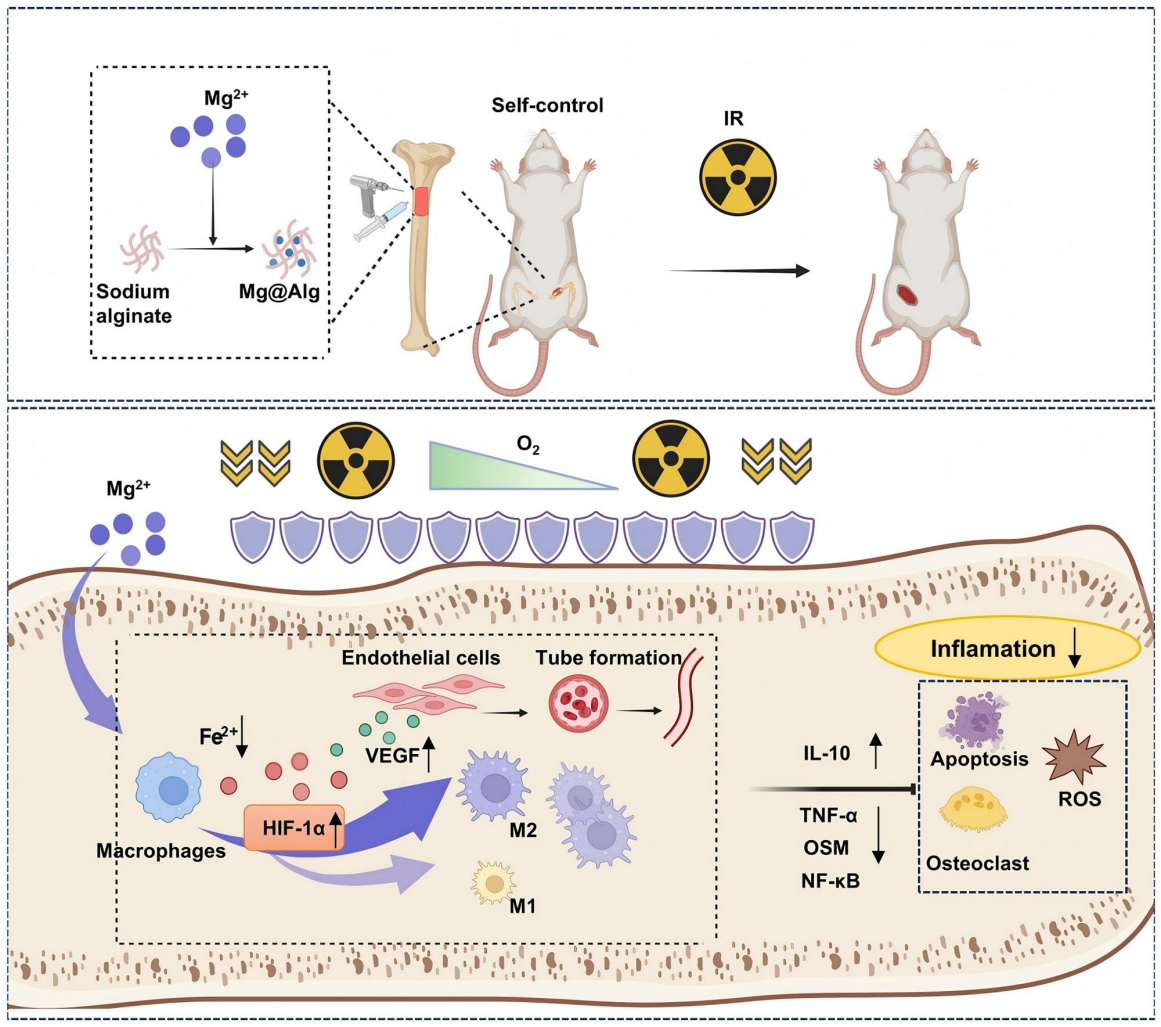
Schematic of Mg^2+^-mediated enhancement of bone radioresistance. Mg@Alg hydrogels sustain high local Mg^2+^ in bone defects. Local Mg^2+^ preconditioning lowers intracellular iron and activates the HIF-1α/VEGF axis, reinforces microvasculature via osteoimmune modulation, and promotes M2-like macrophage polarization. These immune–vascular coupling changes increase radioresistance during peri-radiotherapy, reducing DNA damage, reactive oxygen species (ROS), and osteoclast activation, attenuating inflammation, and reducing RIBI.

To test this hypothesis, we injected Mg^2+^-crosslinked alginate (Mg@Alg) hydrogels into rat femoral defects and compared bone healing in non-irradiated and irradiated settings (Figure 1). We systematically assessed how Mg^2+^ affects key steps in early RIBI, including macrophage polarization and infiltration, microvascular integrity, DNA damage, and osteoclast activation. We further explored irradiation-specific mechanisms of Mg^2+^, focusing on iron-dependent regulation of the hypoxia-inducible factor (HIF) pathway. This work provides a strategy and experimental evidence for using Mg^2+^ to prevent and treat RIBI and informs the translational use of magnesium-based biomaterials for bone protection and repair during radiotherapy.

## Materials and methods

### Cell culture and irradiation

In this experiment, RAW264.7 cells derived from Abelson murine leukemia virus and human umbilical vein endothelial cells (HUVECs) were used. Both cell types were obtained from Guangzhou Xinyuan Biotechnology Co., Ltd (China). RAW264.7 cells were cultured in DMEM, and HUVECs were cultured either in DMEM or macrophage-conditioned medium (CM). The DMEM was supplemented with 10% fetal bovine serum (Thermo Fisher Scientific, USA) and 1% (v/v) penicillin/streptomycin (Thermo Fisher Scientific, USA). Additionally, Magnesium chloride (MgCl_2_) was added to adjust the Mg^2+^ final concentration to 0–40 mM. Groups were named based on the Mg^2+^ concentration: Mg5 (5 mM Mg^2+^), Mg10 (10 mM Mg^2+^), Mg20 (20 mM Mg^2+^) and Mg40 (40 mM Mg^2+^). A control group with an equivalent volume of 1× phosphate-buffered saline (PBS) was also included. CM for HUVECs was prepared by culturing macrophages with varying concentrations of Mg^2+^ for 48 h, after which the medium was collected, filtered for sterilization, and mixed with fresh DMEM containing Mg^2+^ in a 1:1 ratio. Cells were cultured under 5% CO_2_ at 37°C, with the medium being replaced every 2 days. Macrophages were passaged using a cell scraper when they reached 80% confluence, whereas HUVECs were passaged using trypsin digestion at the same confluence level. Irradiation was performed using an X-ray irradiation system (RS-2000 PRO, Rad Source, USA) at a dose of 8 Gy and a dose rate of 1.65 Gy/min, following previously established protocols for cell irradiation [4, 34].

### Cell proliferation and reactive oxygen species measurement

The effect of Mg^2+^ on the proliferation of RAW264.7 macrophages and HUVECs was assessed using a Cell Counting Kit-8 (CCK-8) (Beyotime, China). The inhibition of reactive oxygen species (ROS) production by Mg^2+^ in RAW264.7 macrophages and HUVECs was evaluated using a Reactive Oxygen Species Assay Kit (MAK143-1KT, Sigma-Aldrich, USA).

### Macrophage polarization evaluation by flow cytometry

Macrophages were cultured with varying concentrations of Mg^2+^ for 24, 48 or 72 h and subsequently harvested using a cell scraper. Cells were incubated with CD86-FITC, CD206-APC, or relevant isotype control antibodies (all from Invitrogen, Thermo Fisher Scientific, USA) at 25°C in the dark for 1 h, followed by analysis using a Flow Cytometer (NovoCyte, Agilent Technologies, USA). For irradiated cells, Mg^2+^ pretreatment was followed by irradiation, with subsequent incubation for 24, 48 or 72 h before analysis using the same flow cytometry procedure. Each sample consisted of 1.5 × 10^4^ cells.

### Western blotting

Western blot analysis was performed to assess the protein levels of HIF-1α, HIF-2α, interleukin-10 (IL-10), vascular endothelial growth factor A (VEGFA), bone morphogenetic protein-2 (BMP-2), nuclear factor kappa B (NF-κB), prolyl hydroxylase domain-2 (PHD2), factor-inhibiting hypoxia-1 (FIH1), Von Hippel–Lindau tumor suppression protein (VHL) and regulated on activation normal T expressed and secreted (RANTES) in RAW264.7 macrophages. Following pretreatment with varying concentrations of Mg^2+^ for 48 and 72 h, cells were lysed in RIPA buffer (Thermo Fisher Scientific, USA), and protein concentrations were measured using a BCA Protein Assay Kit (Thermo Fisher Scientific, USA). Samples were denatured, loaded for SDS-PAGE and transferred to PVDF membranes in an ice-water bath. Membranes were blocked with 5% bovine serum albumin and incubated overnight at 4°C with primary antibodies: anti-HIF-1α, anti-HIF-2α, anti-IL-10, anti-VEGFA, anti-BMP-2, anti-RANTES, anti-Phospho-NF-κB p65 (Ser536), anti-PHD2, anti-FIH1 and anti-VHL (all from Affinity USA), with β-actin or GAPDH as control. Results were visualized using an ECL substrate (Thermo Fisher Scientific, USA) and a ChemiDoc XRS+ System (Bio-Rad, USA).

### Quantitative real-time polymerase chain reaction

Quantitative real-time polymerase chain reaction (q-PCR) was employed to investigate the gene expression of HIF-1α, HIF-2α, TNF-α, BMP-2, C–C motif chemokine ligand 5 (CCL5), Oncostatin M (OSM), IL-10, PHD2, FIH1, VHL and VEGFA in RAW264.7 macrophages under different Mg^2+^ conditions. The cells were cultured for 48 h, harvested, centrifuged and transferred for RNA extraction. Gene expression was analyzed using q-PCR, with GAPDH served as an internal control. The primer sequences are listed in Table S1 of the Supporting Data.

### Ferritin detection by enzyme-linked immunosorbent assay and Fe^2+^ supplementation assays

Ferritin levels in RAW264.7 cells after 48 h of Mg^2+^ treatment were measured using a mouse ferritin enzyme-linked immunosorbent assay kit (Mlbio, China), following the manufacturer’s instructions. For Fe^2+^ supplementation, a freshly prepared FeSO_4_ solution (final concentration 30 mM) was added to the Mg20 group and incubated for 6 h [35, 36]. Cells were then collected for downstream assays, including Western blotting, q-PCR, macrophage polarization evaluation by flow cytometry, and tube-formation assay.

### DNA damage assay

DNA damage was assessed using the γ-H2AX immunofluorescence assay kit (Beyotime, China) to evaluate the effects of IR stimulation on macrophages and the protective role of Mg^2+^. MgCl_2_ was added to the macrophage culture medium, and after 48 h, the cells were irradiated and cultured for another 48 h. Following the instructions provided by the kit, DNA damage was observed using a laser confocal microscope (LSM700, ZEISS, Germany).

### Scratch-wound assays

After IR treatment, the HUVECs were seeded into 12-well plates. Once the cells reached 70% confluence, vertical scratches were made across using a 200 µL pipette tip. The plates were washed with 1× PBS to remove the detached cells, and the medium was replaced with CM. Then the plates were moved back into incubator. Images were captured at 0, 24 and 48 h, and the area of the scratch without cells was measured using ImageJ to calculate cell migration rates.

### Transwell migration assay

An 8-µm pore transwell insert was placed in a 12-well plate, and 3 × 10^4^ IR-treated HUVECs were seeded into the upper chamber. Then, 700 µL of CM was added to the lower chamber. Following a 24-h incubation, non-migrated cells remaining on the upper surface of the insert were gently removed, and the insert was washed 2–3 times with PBS. The remaining cells were then fixed with paraformaldehyde and stained with 0.1% crystal violet. After drying, images were captured using a microscope, and cell counts were quantified with ImageJ software.

### Tube-formation assay

The Ceturegel^®^ Matrix High Concentration, Phenol Red-Free, LDEV-Free (Yeasen Biotechnology, China) was diluted 1:2 with serum-free medium and placed on ice. The matrix-coated 96-well plates were incubated at 37°C for 45 min to allow gelation. Subsequently, 2 × 10^4^ IR-treated HUVECs were seeded into each well and incubated with CM for 24 h. Tube formation was observed under a microscope, and the analysis was performed using the Angiogenesis Analyzer plugin in ImageJ.

### Preparation of Mg^2+^-crosslinked hydrogel

MgCl_2_ (CAS: 7786-30-3) and sodium alginate (SA, CAS: 9005-38-3) were obtained from Sigma-Aldrich (USA). For hydrogel preparation, 0.5 g MgCl_2_ and 0.5 g of SA were exposed to UV light for 1 h. Subsequently, the Mg@Alg hydrogels were prepared by magnetic stirring of the powders with 9 g of sterilized deionized water. Mg^2+^-enriched hydrogels containing 2 wt% and 8 wt% MgCl_2_, along with a 5 wt% SA gel (Alg) were prepared simultaneously. All the gels were stored at 4°C.

### Characterization of hydrogels

Following freeze-drying of the Mg@Alg and Alg samples, the characteristic absorption peaks of both hydrogels were identified using Fourier Transform Infrared Spectroscopy (FTIR, Nicolet IS10, USA). Scanning electron microscopy (SEM, ULTRA-55 ZEISS, Germany) was utilized to observe the microstructural differences between the two samples. Compression testing was performed on fresh hydrogels using a Nano Indenter G200 (Agilent, USA) to determine their initial elastic modulus. For the magnesium ion release assay, Mg@Alg hydrogels were immersed in 1 × PBS (pH 7.4) at a concentration of 0.05 g/mL. Released medium (2 mL) was collected at different time points, followed by replacement with fresh buffer to maintain sink conditions. The collected solutions were analyzed for Mg^2+^ concentration using inductively coupled plasma atomic emission spectroscopy (ICP-AES, iCAP 7000, Thermo Fisher Scientific, USA), and a release profile was plotted. The swelling behavior was assessed by measuring the weight change of hydrogels before and after immersion in PBS under the same conditions. In addition, the degradation behavior of Mg@Alg hydrogels was evaluated under various pH conditions for comparison.

### Animal surgery

For animal experiments, 33 male SD rats, 6–8 weeks of age, weighing 200–250 g, were obtained from Guangzhou Viton Lever Co., Ltd (China), and housed at the Jinan University Animal Experimental Center. The study was approved by the Jinan University Animal Welfare and Ethics Committee (approval no. IACUC-20230329-28) and followed ethical and welfare guidelines (Animal Use License No. SYXK (Yue) 2022-0174). Animals were anesthetized with 3% sodium pentobarbital (Sigma-Aldrich, USA) solution (30 mg/kg) via intraperitoneal injection. A longitudinal incision was made on the anterior side of the distal femur to expose the bone. A 2-mm diameter drill was used to create a defect perpendicular to the femoral axis and parallel to the knee joint surface, resulting in a 2-mm deep defect (4 mm × 2 mm × 2 mm). Each rat served as its own control, with defects in both femurs (Supplementary Figure S1A). Mg@Alg was injected into the left femoral defect, using a 50-ml syringe, and Alg was injected into the right femur as a control. The incision was sutured layer by layer, and the rats were maintained at 20°C until they fully recovered. One week post-operation, some rats were anesthetized. Then non-irradiated areas were covered with lead plates, and irradiation was performed using a small animal irradiator (RS-2000 PRO, Rad Source, Germany) at a dose of 20 Gy and a dose rate of 2 Gy/min (Supplementary Figure S1B), following the literature guidelines [4, 37]. Irradiated rats were maintained under normal conditions, with some rats used as non-irradiated controls.

### SEM and energy dispersive spectroscopy characterization

7 days post-operation in rat femoral bone defects (without IR), the rats were euthanized via an intraperitoneal overdose of chloral hydrate (Guangdong Daxiao Chemical Industry Co., Ltd, China). Bilateral femur samples were collected, fixed in 4% paraformaldehyde solution for 48 h, after which the surface soft tissues were removed. The samples were dried, and analyzed using SEM and energy dispersive spectroscopy (EDS) (XFlash 6, Bruker, Germany) to determine the composition and distribution of Mg, Ca and P in the surgical area (Au was not included in the elemental analysis).

### Biocompatibility assessment

Fourteen days post-operation (without IR) and in the pure surgery group (where only the femoral bone defect model was established without hydrogel implantation), rats were euthanized. The heart, liver and kidney samples were obtained, fixed in 4% paraformaldehyde for 48 h, dehydrated and embedded in paraffin. Histological sections were prepared and stained with hematoxylin and eosin (HE) for analysis. Blood samples were collected from the heart 14 days postoperatively and centrifuged at 3000 rpm for 10 min to collect the serum. The serum concentrations of Mg, Cl, Ca and P ions were measured using an automated blood analyzer (Chemray 240, Rayto, China).

### Micro-computed tomography analysis

At 21 days post-operation, rats with and without IR treatment were euthanized (*n *= 5), and the femur samples were collected and fixed in 4% paraformaldehyde. All samples were scanned using micro-computed tomography (CT) (SkyScan1276, Bruker, Germany) with the following parameters: tube current of 200 µA, voltage of 70 kV, resolution of 6.53 µm, exposure time of 350 ms, and a scan angle of 180°. Three-dimensional reconstruction was performed using the NRecon software (version V1.7.4.2, Bruker, Germany). Reconstructed images were analyzed using CT Analyzer software (version 1.20.3.0, Bruker, Germany) to determine parameters such as total volume (TV), bone volume (BV), volume ratio (BV/TV), trabecular number (Tb. N), trabecular thickness (Tb.th) and bone mineral density (BMD).

### Magnetic resonance imaging analysis

On days 0, 3, 7 and 14 post-IR, rats were anesthetized and then fixed in an animal-specific coil. Scanning was performed using a 3.0 T magnetic resonance imaging (MRI) scanner (Bruker, Germany) with conventional axial positioning for T2-weighted imaging. The MRI parameters were as follows: TR/TE = 2500/57 ms, FOV 5 cm × 5 cm; matrix 256 × 256, and slice thickness 2 mm.

### Histological staining

On days 0, 3, 7 and 14 post-IR, rats were euthanized, and bilateral femurs were collected, fixed in 4% paraformaldehyde, and decalcified in 12.5% ethylenediamine tetraacetic acid for 3 weeks (with solution changes every 3 days until the specimen could be easily pierced with a needle). The specimens were then dehydrated in ethanol, embedded in paraffin and sectioned longitudinally into 5-µm-thick slices. Each sample underwent HE, Masson staining and tartrate-resistant acid phosphatase (TRAP) staining according to the instructions provided by the manufacturer (Servicebio, China).

### Immunohistochemistry and immunofluorescence staining

On days 0, 3, 7 and 14 post-IR, paraffin sections of femoral samples were prepared as described in the section “Histological staining”. After dewaxing, antigen retrieval and blocking with 3% H_2_O_2_ and 10% normal goat serum, sections were incubated overnight at 4°C with primary antibodies: rabbit anti-HIF-1α, rabbit anti-HIF-2α, rabbit anti-IL-10, rabbit anti-VEGFA, rabbit anti-CCL5, rabbit anti-TNF-α, rabbit anti-Caspase-3, rabbit anti-BMP-2, rabbit anti-Transforming growth factor-β (TGF-β), and rabbit anti-OCN for immunohistochemistry (all antibodies from Affinity USA). Following secondary antibody incubation, DAB staining was performed using a DAB kit (Beyotime, China). For immunofluorescence, sections were incubated with rabbit anti-CD68 and rabbit anti-CD206 primary antibodies at 4°C for 15 h, followed by secondary antibody incubation for 1 h at 25°C. 4',6-diamidino-2-phenylindole (DAPI) was used for nuclear staining. CD31, endomucin (Emcn), α-smooth muscle actin (α-SMA), γ-H2AX and NF-κB (all antibodies from Invitrogen, Thermo Fisher Scientific, USA) immunofluorescence staining was also performed, and sections were observed under a laser confocal microscope.

### Statistical analysis

Data were analyzed using SPSS 25.0 and GraphPad Prism 9.0 software, with results presented as mean ± standard deviation (SD). Statistical significance was assessed using one-way analysis of variance in the *in vitro* experiments, followed by Tukey’s multiple comparison test. While paired *t*-tests were applied to assess the differences between groups in the *in vivo* experiments. A two-sided significance level of α = 0.05 was used, with *P*-values < 0.05 were considered statistically significant. Statistical significance is indicated by asterisks (*P < 0.05, **P < 0.01, ***P < 0.001 and ****P < 0.0001). “ns” denotes no statistical difference.

## Results

### Mg@Alg increases local Mg^2+^ concentration during the peri-radiotherapy period, reducing bone loss post-IR

**Figure 2.**
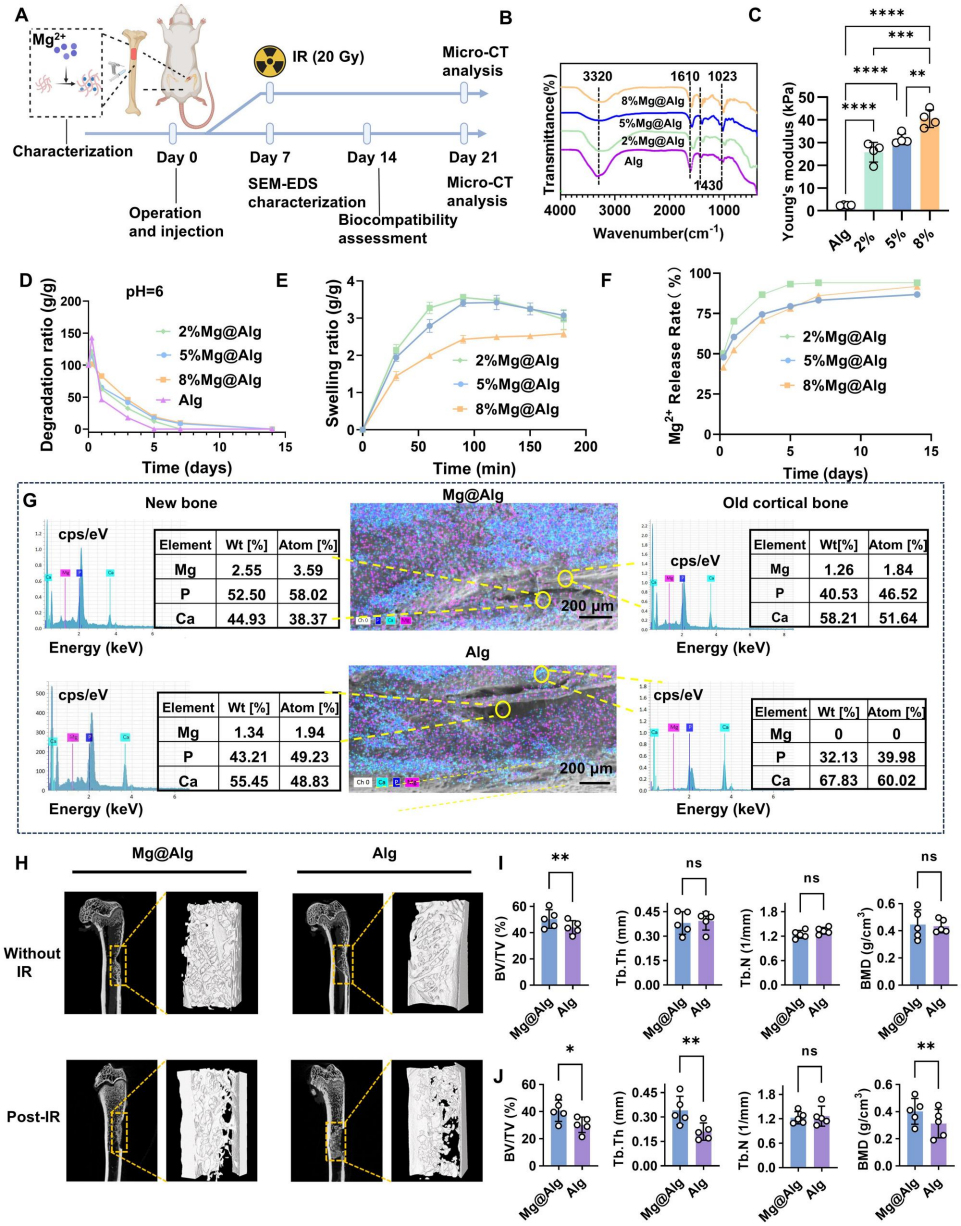
Characterization and biological response of the hydrogels. (**A**) Schematic diagram of Mg@Alg preparation and rat experiments under normal and IR conditions. (**B**) FTIR spectra of hydrogels in the dry state. (**C**) Young’s modulus of the hydrogels (*n *= 4). (**D**) Degradation behavior of Mg@Alg at pH = 6 (*n *= 3). (**E**) Swelling behavior of Mg@Alg over 14 days (*n *= 3). (**F**) Cumulative release Mg^2+^ from hydrogels *in vitro* (*n *= 3). (**G**) Representative SEM-EDS images of 5% Mg@Alg and the control group in and around the defects at 7 days post-operation. (**H**) Micro-CT images of rat femora at 21 days post-operation with or without IR. Measurements of BV/TV, Tb.Th, Tb.N, BMD at 21 days post-operation (**I**) without or (**J**) with IR (*n *= 5). Data are expressed as mean ± SD. n.s. *P *> 0.05, *P < 0.05, **P < 0.01, ***P < 0.001, ****P < 0.0001. No significant differences between the other groups.

In this study, we synthesized biodegradable Mg^2+^-enriched hydrogels designed to efficiently and sustainably maintain elevated Mg^2+^ concentrations at surgical defects *in vivo* (Figure 2). Crosslinking between Mg^2+^ and carboxyl groups (–COO^−^) in SA led to a marked decrease in the intensity of the carboxylate asymmetric stretching band at 1600–1650 cm^−1^, and a shift to lower wavenumbers in the FTIR spectra (Figure 2B). Increasing the Mg^2+^ content enhances the degree of crosslinking within the hydrogel network, leading to several property modifications, such as reduced fluidity, increased Young’s modulus, and a slower degradation rate (Figure 2C–E and Supplementary Figure S2). We selected the 5% Mg@Alg hydrogel for subsequent animal experiments based on its suitable degradation profile, which releases approximately 90% of Mg^2+^ into the surrounding environment within 2 weeks (Figure 2F). Its slight fluidity also confers good injectability into bone defects. Hereafter, the 5% formulation is referred to as Mg@Alg.

We injected the Mg@Alg hydrogel into the left femoral defects in rats. One week post-operation, an increase in magnesium content was observed on the surface of both the newly formed bone and adjacent cortical bone, beyond baseline levels (Figure 2G). Concurrently, the experimental group exhibited a statistically significant increase in the Mg/Ca atomic ratio (Supplementary Figure S3), accompanied by a decrease in the calcium ion percentage in bone areas. Systemically, local application of Mg@Alg caused no structural damage to major organs (heart, liver, kidneys) (Supplementary Figure S4A), and did not alter serum Mg, Ca, and *P* levels (*P *> 0.05) (Supplementary Figure S4B), combined with the weight changes in rats (Supplementary Figure S4C), indicating good biocompatibility.

At 21 days post-operation, uniform trabecular structures were visible at the defect sites in both femurs (Figure 2H). BV/TV in the Mg@Alg group was higher than that in the control group (*P *< 0.01), with no significant differences in Tb.N, Tb.Th or BMD (Figure 2I). However, when IR was administered 1 week post-operation, significant differences in bone mass and quality emerged between groups at 14 days post-IR (21 days post-operation). In the control group, bone remodeling showed features of damage, with irregular, fragmented trabeculae suggestive of potential necrosis, whereas the Mg@Alg group displayed more organized, continuous trabeculae. Quantitatively, BV/TV was higher in the Mg@Alg group (40.69% ± 7.82%) than in controls (30.27% ± 5.69%) (*P *< 0.05), and Tb.Th and BMD were also higher, indicating improved BV and mineralization. Conversely, Tb.N was also high in controls, consistent with smaller, weaker and more disorganized trabeculae (Figure 2J).

**Figure 3.**
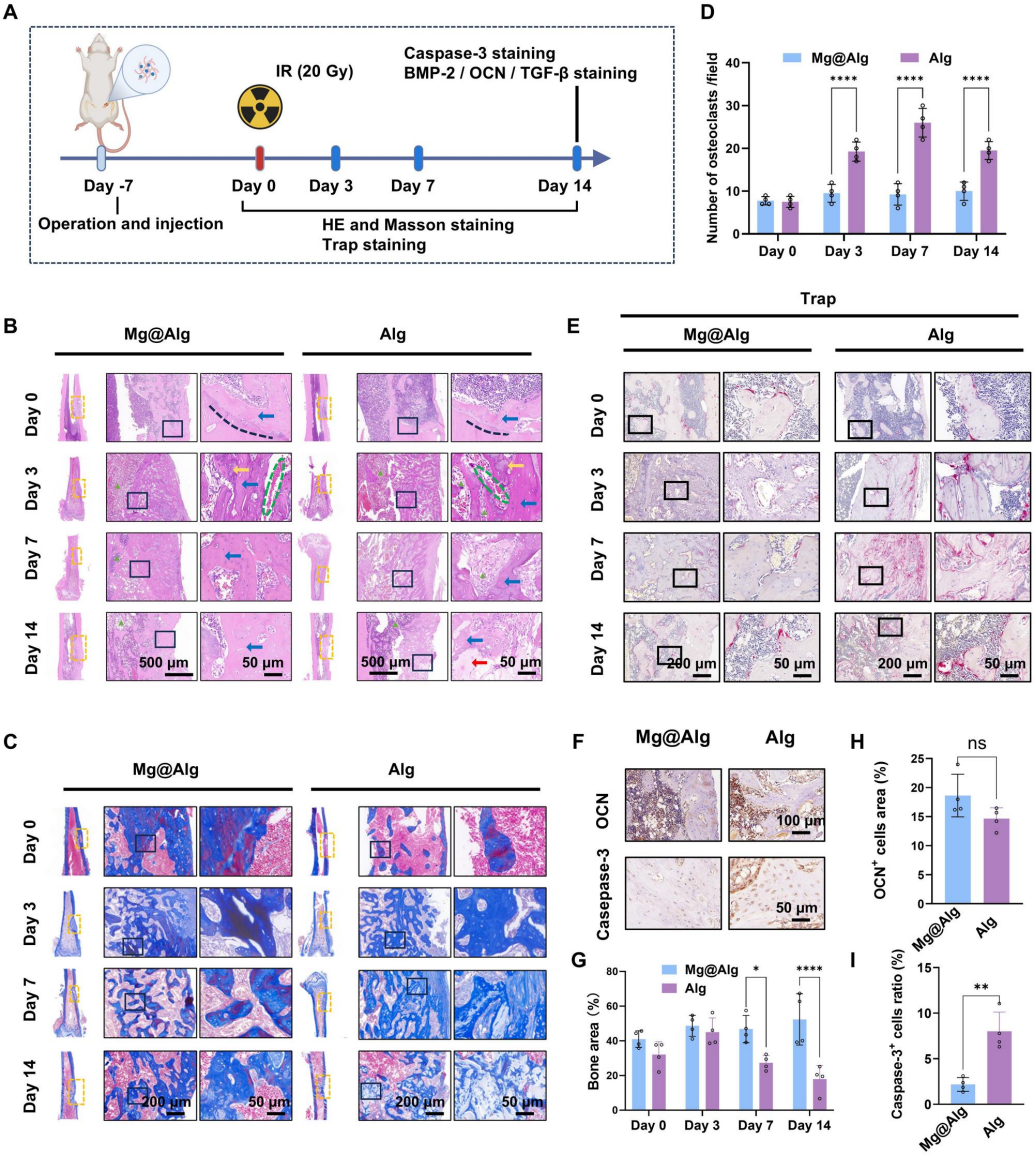
Mg@Alg alleviated bone loss and reduced necrotic bone formation post-IR. (**A**) Schematic diagram of histological staining *in vivo*. Representative images of (**B**) HE staining, (**C**) Masson staining and (**E**) TRAP^+^ osteoclasts in the defects in rats post-IR. The dotted boxes indicate the defect sites; the dashed lines represent new osteoid lines; the triangles represent bone marrow adipogenesis; the blue arrows represent newly formed bone; the yellow arrows represent undegraded hydrogels; the dashed rings represent microvascular congestion, and the red arrows denote areas of necrotic tissue. (**D**) Quantitative analysis of trap^+^ post-IR (*n *= 4). (**F**) Representative images of OCN and caspase-3 in defects at 14 days post-IR. Quantitative analysis of trabecular area in (**G**) Masson staining, (**H**) OCN^+^ and (**I**) Caspase-3^+^ post-IR (*n *= 4). Data are expressed as mean ± SD. n.s. *P *> 0.05, *P < 0.05, **P < 0.01, ****P < 0.0001. No significant differences between the other groups.

Based on these observations, we established the time point of IR (7 days post-operation) as the baseline, and investigated the effects of Mg^2+^ on femoral defects following radiation (Figure 3A). In the early post-IR phase, residual hydrogel fragments were still detectable (Figure 3B). Bone marrow adipogenesis was observed on days 3, 7 and 14 and increased over time; by day 14, the Mg@Alg group exhibited fewer adipose vacuoles within newly formed bone than controls (Figure 3B and C). Masson staining on IR day 3 revealed prominent blue and dark red staining in the Mg@Alg group, indicating active bone repair and mineralization, whereas the control group exhibited reduced mature bone tissue and increased fibrous tissue (Figure 3C). On days 7 and 14, the control group displayed necrotic tissue areas and fibrous connective tissue on the bone surface, with faint blue staining and irregular patches, suggesting abnormal mineralization and necrosis, and bone mass was significantly lower than in the Mg@Alg group (*P *< 0.05) (Figure 3G).

Consistent with Masson’s results, TRAP^+^ staining showed divergent osteoclast activation trends between groups (Figure 3D and E). Over time, the number of osteoclasts gradually increased in the control (Alg) group, whereas this rise was slower with Mg@Alg. Notably, on IR day 14, osteoclast activation at the edge of the bone marrow cavity in non-surgical regions of irradiated bone was also reduced in the Mg@Alg group (Supplementary Figure S5). The pro-apoptotic marker caspase-3 was strongly expressed in newly formed bone in controls on day 14, whereas viable (non-apoptotic) osteocytes predominated in Mg@Alg (Figure 3F and I). Meanwhile, immunohistochemical staining for bone formation markers (OCN, BMP-2 and TGF-β) in defect sections on IR day 14 showed no significant differences between groups (Figure 3F and H and Supplementary Figure S6). Together with the micro-CT findings, these data indicate that under irradiation, Mg@Alg confer early bone-preserving benefits, likely by dampening osteoclast activation and mitigating radiation-induced cellular damage, thereby enhancing anti-resorptive capacity post-IR.

### Mg@Alg increases the M2-like macrophage proportion and mitigates inflammation post-IR

**Figure 4.**
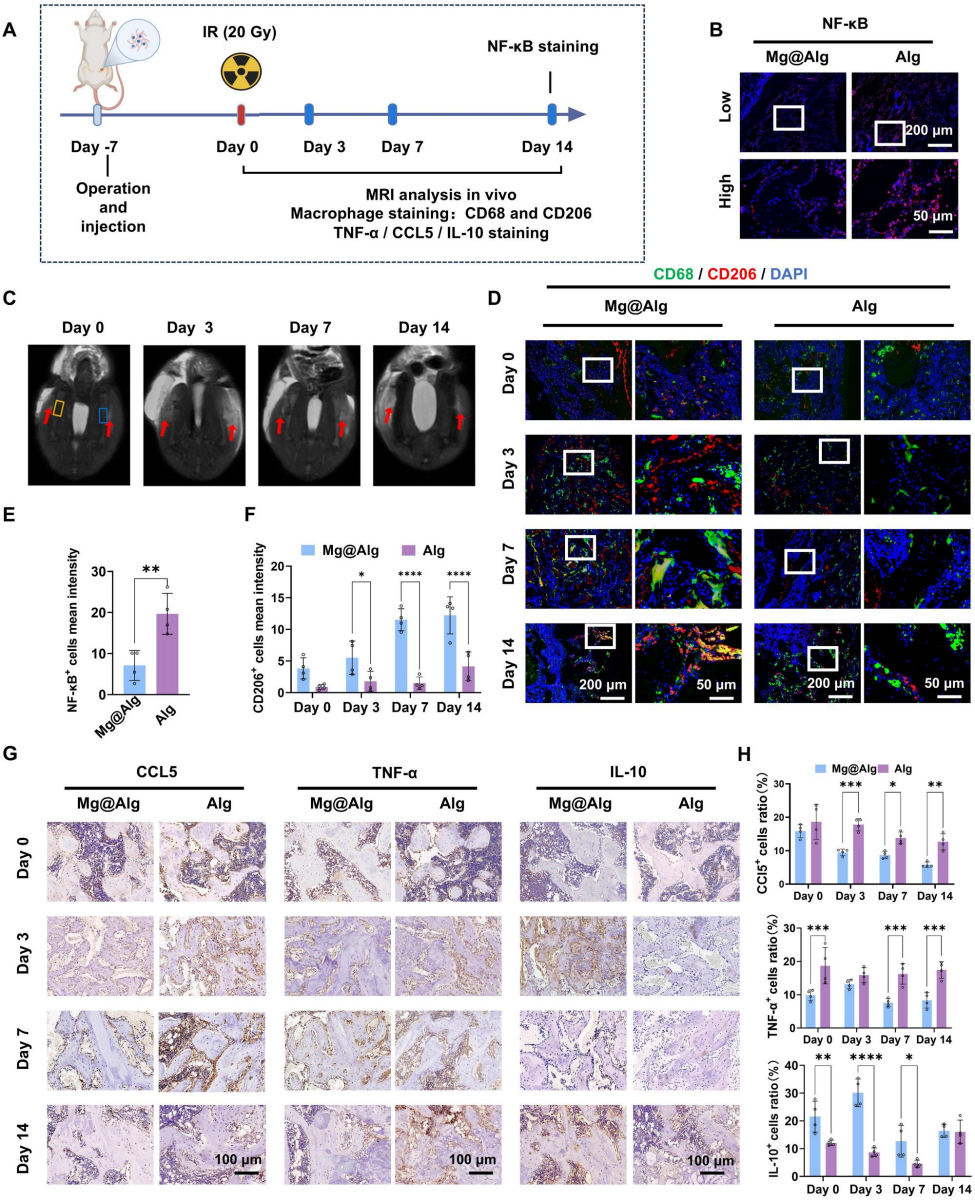
Mg@Alg increases the proportion of M2-like macrophages and mitigates inflammation post-IR. (**A**) Schematic diagram of the workflow for detecting macrophage infiltration and inflammatory response post-IR. (**B**) Representative images of NF-κB in defects at 14 days post-IR. (**C**) Representative images of T2-weighted MRI of the femur and surrounding soft tissues in rats. Boxes indicate the femoral surgical sites, with the right for Mg@Alg and the left for control; arrows indicate areas of inflammatory edema. (**D**) Representative immunofluorescent images of CD68 (green) and CD206 (red) illustrating the presence of macrophages. Quantitative analysis of (**E**) NF-κB^+^ and (**F**) CD206^+^ (M2) cells (*n *= 4). (**G**) Representative images and (**H**) quantitative analysis of CCL5, TNF-α and IL-10 post-IR (*n *= 4). Data are mean ± SD. *P < 0.05, **P < 0.01, ***P < 0.001, ****P < 0.0001. No significant differences between the other groups.

Building on the observed bone-preserving effects and reduced osteoclast activation, we next examined the inflammatory microenvironment of bone defects *in vivo* during the peri-radiotherapy period (Figure 4A). T2-weighted MRI of the femur and surrounding soft tissues was used to evaluate the edema (Figure 4C). On IR day 0, a slightly hyperintense signal was observed at the surgical site. By IR day 3, signal intensity had increased, indicating higher tissue water content consistent with exacerbation of local inflammation post-IR. The edema signal was stronger in the Mg@Alg group than in controls at this time point. From IR days 3–14, the soft-tissue edema signal in the Mg@Alg group gradually decreased, whereas it continued to increase in controls; by day 14, local edema was significantly greater in controls than in the Mg@Alg group.

Immunofluorescence staining of defect sections using CD68 (general macrophage marker) and CD206 (M2 marker) showed marked macrophage infiltration shortly after IR, with a predominance of CD68^+^ CD206^+^ (M2) cells in the Mg@Alg group (Figure 4D and F). In contrast, the Alg control group contained fewer M2-like cells and more CD68^+^CD206^−^ macrophages. Immunohistochemical results further showed that within 14 days post-IR, TNF-α and CCL5 expression were lower in Mg@Alg than in controls, whereas IL-10 was higher (Figure 4G and H). Moreover, on day 14, expression of NF-κB—a central transcription factor in inflammatory responses—was significantly lower in the Mg@Alg group (Figure 4B and E). Together, these findings indicate that post-IR, Mg@Alg accelerates pro-regenerative immune polarization while attenuating overall inflammation in the defect microenvironment, which may rapidly correct the bone imbalance caused by IR injury and contribute to the early bone-preserving effects.

### Mg@Alg promotes microvascular regeneration and maturation post-IR

**Figure 5.**
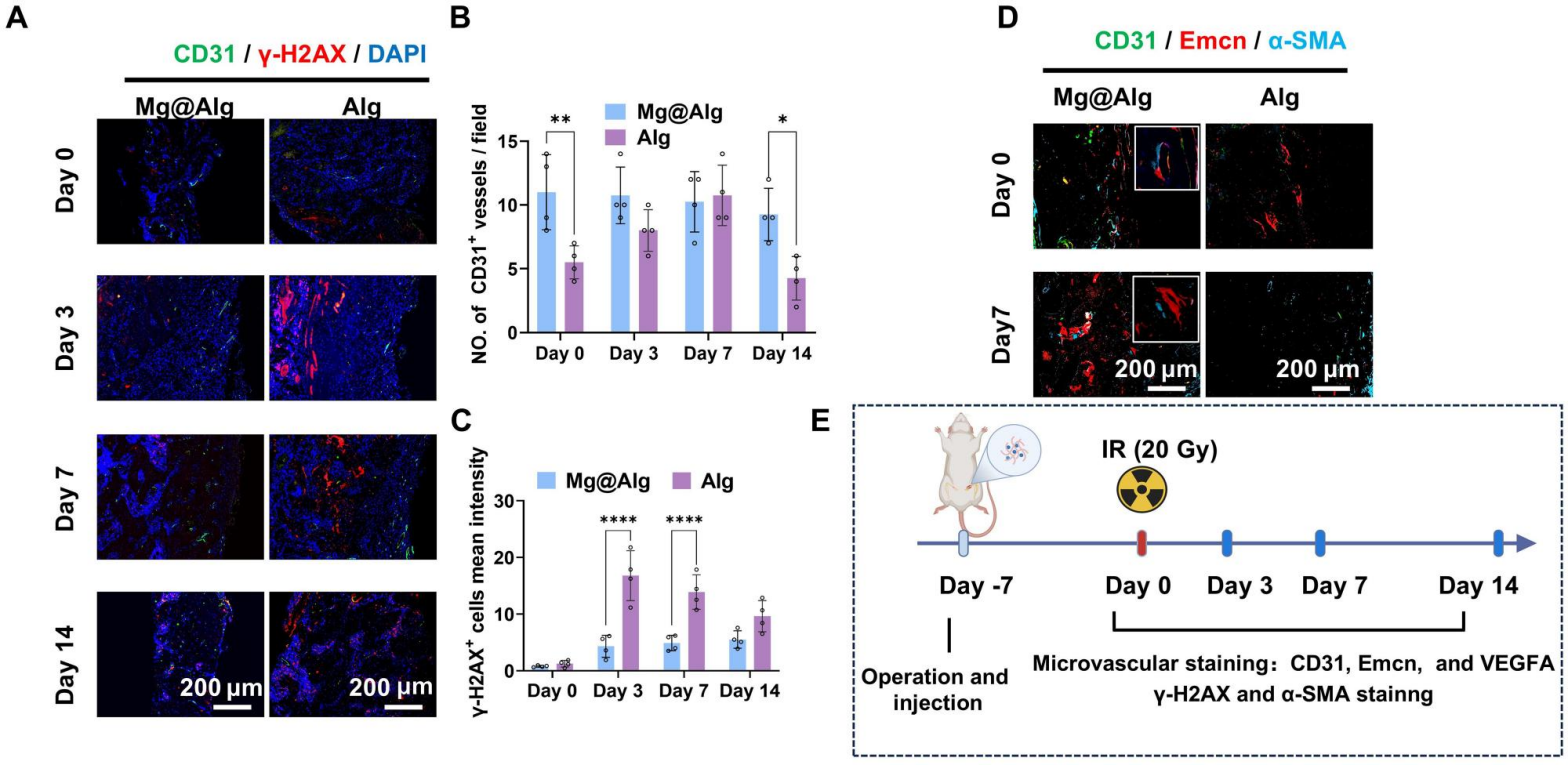
Mg@Alg enhances microvasculature post-IR. (**A**) Representative immunofluorescent images of CD31 and γ-H2AX post-IR. Quantitative analysis of (**B**) CD31^+^ microvascular structures and (**C**) γ-H2AX^+^ cells (*n *= 4). (**D**) Representative immunofluorescent images of CD31, Emcn and α-SMA at day 0 and day 14 post-IR. The box indicates a magnified view of CD31 Emcn^+^ endothelial cells with α-SMA^+^ cells. (**E**) Schematic diagram of the workflow for microvasculature staining. Data are mean ± SD. *P < 0.05, **P < 0.01, ****P < 0.0001. No significant differences between the other groups.

We evaluated microvascularization at serial time points post-IR. CD31 immunofluorescence revealed a higher density of CD31^+^ microvessels in the Mg@Alg group over the first 14 days post-IR (*P *< 0.05) (Figure 5A and B). The DNA damage marker γ-H2AX was negative or weakly positive in regions adjacent to CD31^+^ microvessels, implying reduced DNA damage in areas with richer microvasculature. Quantitatively, the Mg@Alg group exhibited a lower γ-H2AX signal density than controls across time points (Figure 5A and C). To assess vascular phenotype and maturation, we performed CD31/Emcn/α-SMA triple-immunofluorescence confocal imaging. The Mg@Alg group displayed increased CD31^+^/Emcn^+^ microvessels with more preserved morphology, expanded Emcn^+^ neovessel hotspots and perivascular α-SMA^+^ cells surrounding endothelial structures (Figure 5D). Consistently, VEGFA expression at day 7 post-IR was significantly higher in the Mg@Alg group (Supplementary Figure S7), supporting a pro-angiogenic effect.

### Mg^2+^ enhances the radioresistance of RAW264.7 macrophages, while IR augments Mg^2+^ responsiveness

**Figure 6.**
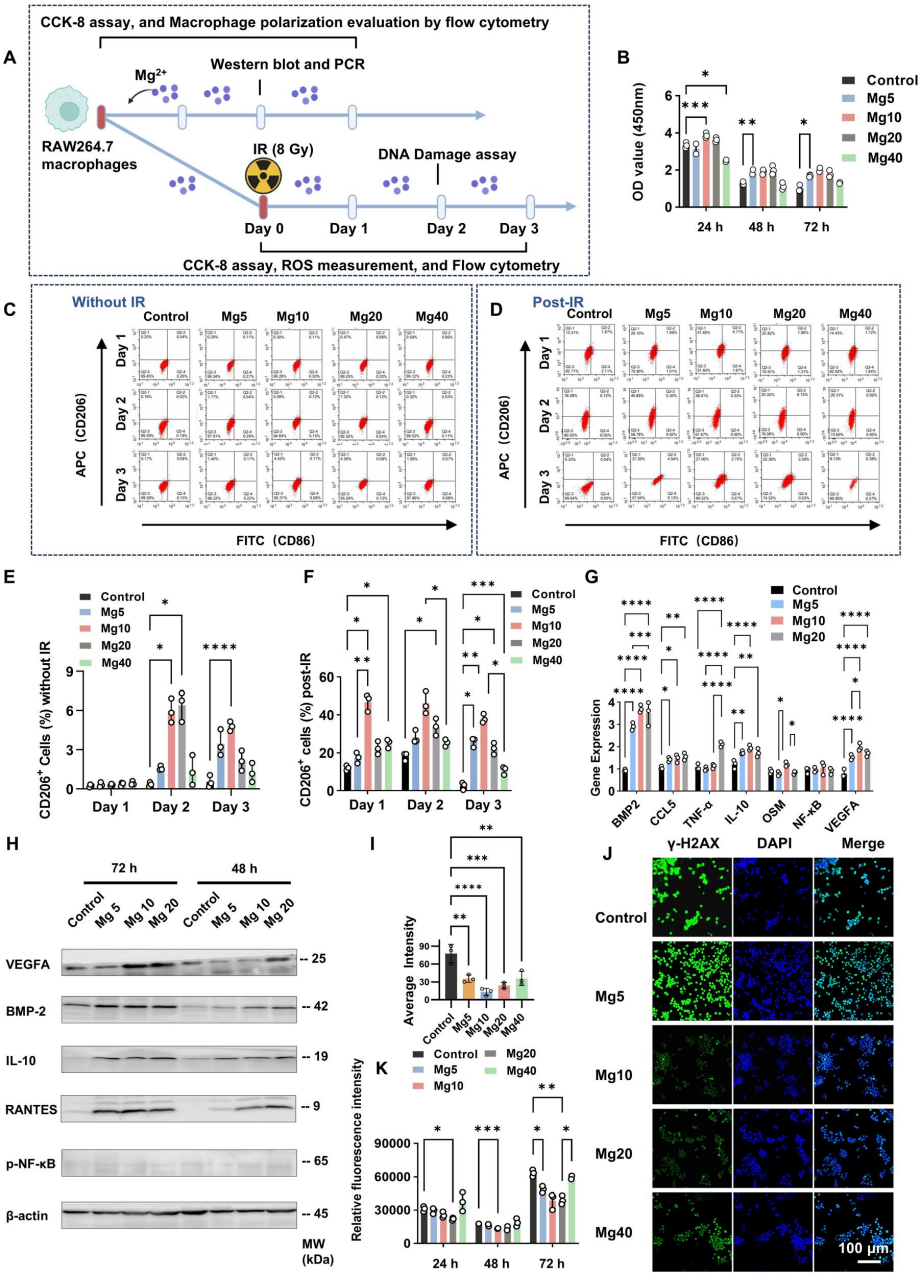
Mg^2+^ enhances the radioresistance of RAW264.7 macrophages. (**A**) Schematic diagram illustrating Mg^2+^ treatment of RAW 264.7 macrophages during the peri-radiotherapy period. (**B**) Effects of 48-hour Mg^2+^ pretreatment on the cell proliferation under IR condition (*n *= 3). Regulation of macrophage polarization by Mg^2+^ under (**C**) normal conditions and (**D**) IR conditions (M1: CD86-FITC, M2: CD206-APC). Percentage of CD206^+^ cells under (**E**) normal conditions and (**F**) IR conditions (*n *= 3). (**G**) q-PCR and (**H**) Western blot analysis of IL-10, BMP-2, NF-κB, TNF-α, CCL5, OSM, RANTEs and VEGFA (*n *= 3). (**I**) Average fluorescence intensity and (**J**) representative immunofluorescent images of γ-H2AX^+^ post-IR (*n *= 3). (**K**) Effects of Mg^2+^ pretreatment on ROS production post-IR (*n *= 3). Data are expressed as mean ± SD. *P < 0.05, **P < 0.01, ***P < 0.001, ****P < 0.0001. No significant differences between the other groups.

We next assessed the direct effects of Mg^2+^ (0–40 mM) on RAW264.7 macrophages with or without IR (Figure 6). Compared with non-irradiated conditions, Mg^2+^ in irradiated cultures showed earlier pro-proliferative responses and stronger M2-polarizing effects, but with a narrower biocompatible concentration range. Without IR, Mg^2+^ up to 40 mM did not significantly change proliferation over 72 h (Supplementary Figure S8A). By contrast, after IR, pro-proliferative effects emerged by 48 h in a dose-dependent manner (Supplementary Figure S8B and C and Figure 6D). Flow cytometry showed that IR markedly enhanced Mg^2+^-driven M2-like polarization: with 48 h Mg^2+^ preconditioning followed by IR, the Mg10 group reached 41.6% M2-like cells at 24 h post-IR (Figure 6C–F). Across the first 3 days post-IR, 5–10 mM Mg^2+^ consistently increased the M2-like fraction and mitigated IR-induced cytotoxicity (Figure 6D and F and Supplementary Figure S9B). However, this heightened responsiveness to Mg^2+^ under IR was accompanied by reduced biocompatibility, as 40 mM decreased viability.

At the transcriptional and protein levels, 5–20 mM Mg^2+^ upregulated pro-repair and pro-angiogenic/anti-inflammatory mediators, including VEGFA, BMP-2, IL-10 and CCL5 (RANTES), without activating NF-κB signaling or increasing osteoclast-associated genes such as OSM; notably, 10 mM Mg^2+^ did not elevate TNF-α (Figure 6G and H). Functionally, Mg10 group reduced IR-induced DNA damage, as indicated by lower γ-H2AX at 48 h post-IR; higher Mg^2+^ levels weakened this protection (Figure 6I and J). Within 24 h post-IR, 5–20 mM Mg^2+^ also limited ROS more effectively (Figure 6K). Together, these data indicate that Mg^2+^ mitigates IR-induced injury and enhances macrophage radioresistance, while IR increases cellular responsiveness to Mg^2+^.

### Mg^2+^ alleviates IR-induced damage in HUVECs under immune-conditioned cues

**Figure 7.**
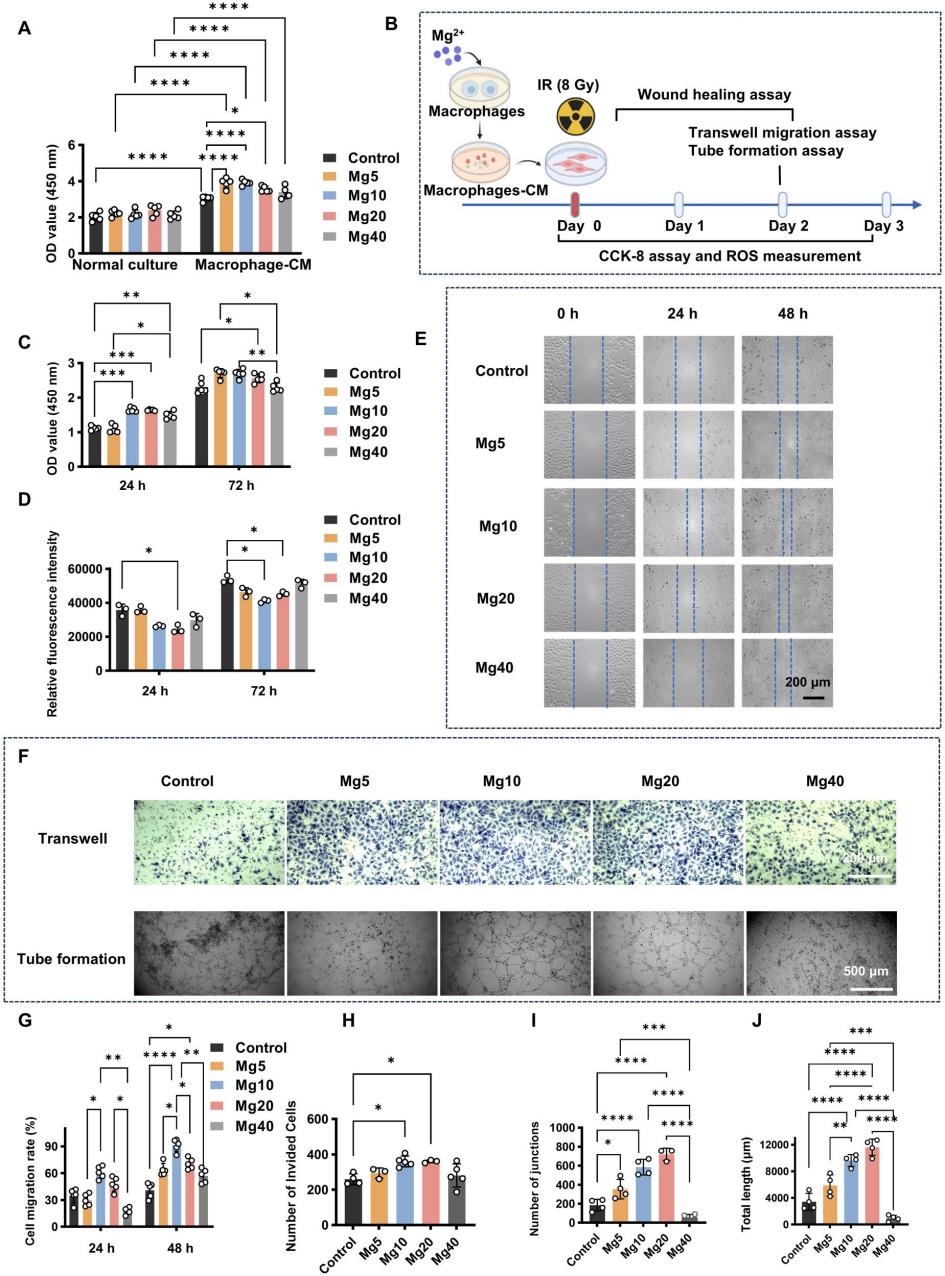
Mg^2+^ modulates HUVEC responses post-IR under macrophage-conditioned cues. (**A**) Effects of Mg^2+^ on the cell proliferation of HUVECs cultured in DMEM and macrophage—CM (*n *= 5). (**B**) Schematic diagram illustrating Mg^2+^ treatment of HUVECs in macrophage-CM during the peri-radiotherapy period. (**C**) Effects of Mg^2+^ on cell proliferation (*n *= 5) and (**D**) ROS production (*n *= 3) in HUVECs post-IR. (**E**) Representative scratch-wound images post-IR. (**F**) Representative transwell migration and tube-formation images of HUVECs at 24 h post-IR. (**G**) Calculation of migration rate of HUVECs in the scratch-wound assay (*n *= 5). (**H**) Quantifications of invaded cells in transwell assay (*n *= 6). Quantifications of (**I**) the number of junctions and (**J**) total length in tube formation (*n *= 4). Data are expressed as mean ± SD. *P < 0.05, **P < 0.01, ***P < 0.001, ****P < 0.0001. No significant differences between the other groups.

We established an HUVEC IR model to assess angiogenic capacity. We first compared HUVEC proliferation with or without CM. Mg^2+^ alone did not significantly increase HUVEC counts. Whereas in the presence of CM, Mg^2+^ significantly promoted proliferation (*P *< 0.05), indicating that immune-conditioned cues support endothelial function (Figure 7B); thus, CM was used in all subsequent HUVEC assays (Figure 7A). After IR, 5–20 mM Mg^2+^ increased HUVEC counts compared with controls and more effectively limited ROS (Figure 7C and D). Scratch-wound and Transwell assays showed faster migration in Mg10 and Mg20 groups (Figure 7E–H). In tube-formation assays 12 h post-IR, extensive cell death limited network formation in the control, with only sparse nodes observed, whereas the Mg10 and Mg20 groups maintained relatively intact networks (Figure 7F, I and J). Together, Mg^2+^ (notably 10 mM), combined with macrophage-derived factors (e.g. VEGFA demonstrated in the section “Mg^2+^ enhances the radioresistance of RAW264.7 macrophages, while IR augments Mg^2+^ responsiveness”) in CM, supports HUVEC survival, migration and network formation, thereby alleviating IR-induced endothelial damage.

### Iron-dependent PHD2/HIF-1α modulation by Mg^2+^ promotes M2-like polarization and pro-angiogenic responses under IR conditions

**Figure 8.**
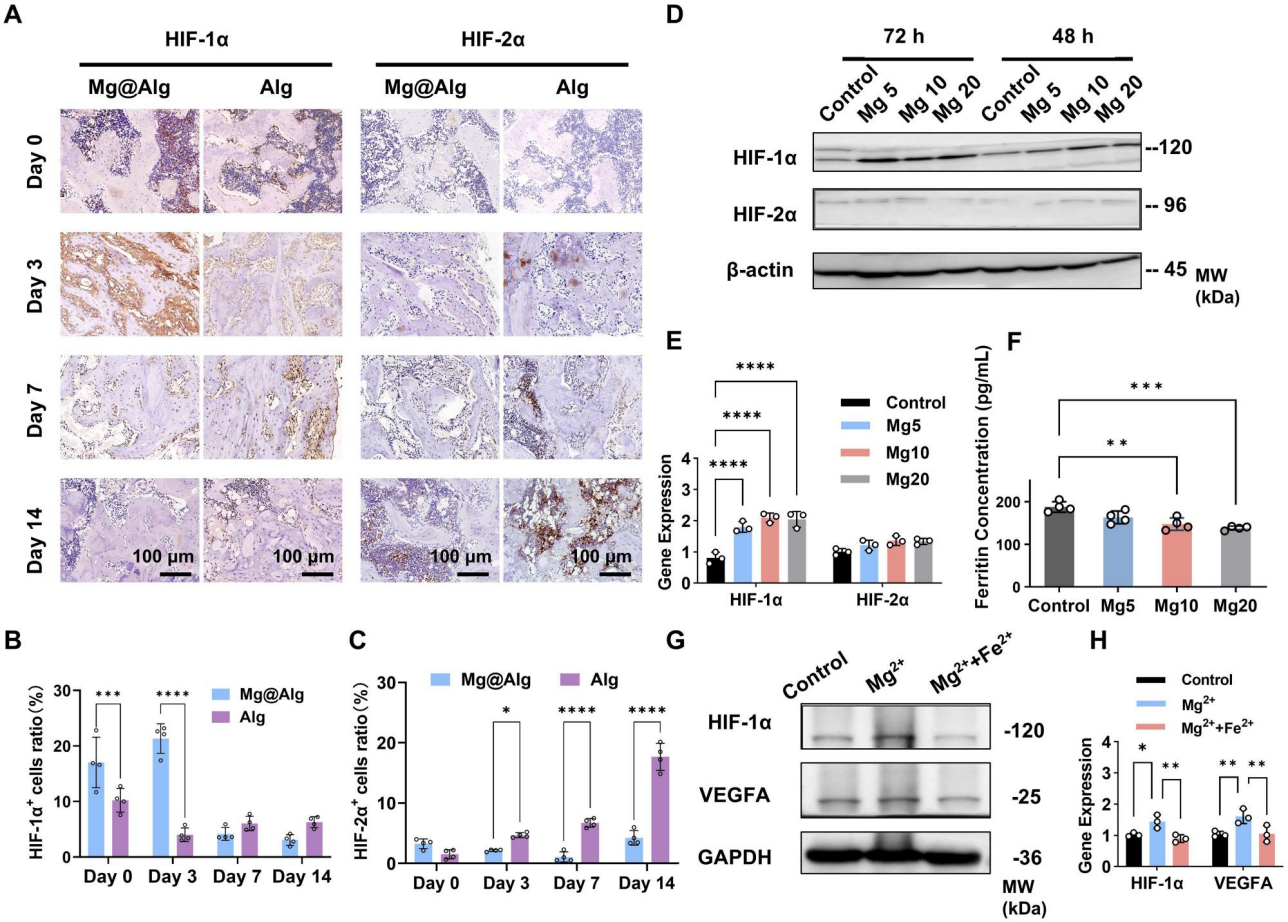
Mg^2+^ enhances HIF-1α stability, and this effect is reversible by Fe^2+^. (**A**) representative immunohistochemical images of HIF-1α and HIF-2α in defects at 0, 3, 7 and 14 days post-IR (*n *= 4). quantitative analysis of (**B**) HIF-1α and (**C**) HIF-2α. (**D**) Western blot and (**E**) q-PCR analysis of HIF-1α and HIF-2α in RAW264.7 macrophages (*n *= 3). (**F**) Ferritin levels in RAW 264.7 cells following 48 h of Mg^2+^ pretreatment (*n *= 4). (**G**) Western blot and (**H**) q-PCR analysis showing that Fe^2+^ supplementation reverses Mg^2+^-associated changes in HIF-1α and VEGFA. Data are mean ± SD. *P < 0.05, **P < 0.01, ***P < 0.001, ****P < 0.0001. No significant differences between the other groups.

To explain the stronger pro-angiogenic and M2-polarizing effects of Mg^2+^ under IR conditions, we focused on the HIF axis, given the hypoxic feature of irradiated bone areas. HIF-1α/2α are major upstream regulators of VEGFA. *In vivo*, within 14 days post-IR, HIF-1α and HIF-2α expression showed time-dependent changes (Figure 8A–C). Compared with controls, Mg@Alg induced an earlier, larger rise in HIF-1α, while HIF-2α was not significantly upregulated and was lower at some time points. In RAW264.7 cells, 5–20 mM Mg^2+^ increased the stability of HIF-1α mRNA and protein, with no change in HIF-2α (*P *> 0.05) (Figure 8D and E).

To probe upstream regulation, we examined intracellular ferritin and HIF-related proteins (PHD2, FIH1, VHL). Mg^2+^ (0–20 mM) reduced ferritin dose-dependently (Figure 8F), and 20 mM pretreatment blunted the 24 h IR-induced ferritin rise (*P *< 0.05, Supplementary Figure S10A). In parallel, 20 mM Mg^2+^ downregulated PHD2 and VHL without detectable changes in FIH1 (Supplementary Figure S10B and C). Fe^2+^ supplementation reversed Mg^2+^-driven changes in HIF-1α, VEGFA and PHD2 (Figure 8G and H and Supplementary Figure S10B and C). Functionally, Fe^2+^ significantly attenuated the Mg^2+^ (20 mM)-induced increase in the M2-like macrophage fraction under IR conditions, diminished Mg^2+^-supported viability and tube formation and increased cytotoxicity versus the control (Supplementary Figure S10D–I). Collectively, our data raise the possibility that Mg^2+^ alters intracellular iron handling to downregulate PHD2 and stabilize HIF-1α, thereby promoting M2-like polarization and pro-angiogenic responses under IR conditions.

## Discussion

The pathological microenvironment may alter the expression and function of drug targets, thereby changing drug mechanisms and efficacy [28–30]. Although magnesium is well known to promote osteogenesis under normal conditions [18], locally released Mg^2+^ produced only limited short-term osteogenic gains in our study, yet significantly reduced bone loss and apoptosis after irradiation. The early inflammatory milieu in irradiated bone critically influences the progression and outcomes of RIBI. Our integrated data suggest that peri-radiotherapy Mg^2+^ primarily enhances the microvascularization while reprogramming osteoimmune responses. These actions restrain osteoclast activation, attenuate inflammatory injury, and facilitate the restoration of bone homeostasis.

The HIF pathway is the central system for sensing and adapting to hypoxia, promoting angiogenesis, maintaining redox balance, and supporting cell survival [38, 39]. Fe^2+^ downregulates the HIF-α subunit and decreases its transcriptional activity via HIF hydroxylases (PHDs and FIH) [40], and irradiation can disrupt iron metabolism [32, 33]. Divalent cations such as Zn^2+^, Ca^2+^ and Mg^2+^ may also influence cellular iron handling [41]. In our study, ferritin rose early after IR; with Mg^2+^ intervention, ferritin decreased in a dose-dependent manner, accompanied by PHD2 downregulation and HIF-1α upregulation. Fe^2+^ supplementation reversed Mg^2+^ effects on HIF pathway, and weakened Mg^2+^-driven M2-like polarization and endothelial tube formation under IR. These findings support an iron-dependent PHD2/HIF-1α mechanism for Mg^2+^.

In our study, the cells were more sensitive to Mg^2+^ under IR conditions: Mg^2+^ improved viability and limited ROS earlier, but showed a sharper biphasic dose response. In irradiated settings, <20 mM Mg^2+^ promoted M2-like polarization, whereas >20 mM reduced benefits or became detrimental. By contrast, in non-irradiated contexts, toxicity typically appears above 60 mM [42, 43]. IR-induced homeostatic imbalance may contribute to this shift: iron dysregulation sensitizes PHD2/HIF-1α signaling to Mg^2+^, while IR-related changes in membrane permeability and ion channels [44, 45] may narrow the therapeutic window. Beyond stabilizing HIF-1α via PHD2 downregulation, Mg^2+^ was associated with reduced NF-κB activity and ROS in this study, partially mirroring effects reported for hypoxia mimetics like cobalt chloride [46] and deferoxamine [47], potentially with a more favorable safety profile, but their comparative efficacy were not assessed.

HIF-1α and HIF-2α showed divergent responses here: Mg^2+^ increased HIF-1α stability but did not affect HIF-2α. *In vivo*, Mg@Alg induced an earlier and stronger HIF-1α rise; in controls, HIF-2α increased by day 14, coinciding with necrotic changes in bone areas. Literature indicates that HIF-2α is more complexly regulated by iron metabolism—its stability depends on PHDs, while its translation can be selectively inhibited by iron regulatory protein 1 [48]. HIF-1α mainly mediates acute hypoxia responses, whereas HIF-2α accumulates later [49] and may act as a negative regulator in irradiated bone [16], consistent with our observations. Although our data support a model in which under IR-induced iron dysregulation, Mg^2+^ modifies cellular iron handling to lower PHD2 and thereby stabilize HIF-1α, our evidence is largely at the expression and phenotypic levels. To substantiate this mechanism, future studies should directly quantify PHD2 enzymatic activity, assess HIF-1α proline hydroxylation and VHL binding, and incorporate appropriate positive controls. These efforts will more rigorously define the Mg^2+^- HIF axis, elucidate how Mg^2+^ interfaces with HIF signaling, and evaluate the feasibility of Mg^2+^ as a hypoxia-mimetic strategy.

Studies have highlighted the importance of macrophage activation states in tissue repair and regeneration [50]. In our study, Mg^2+^ promoted M2-like polarization, with a stronger effect after IR, suggesting possible endogenous compensatory cues. Efferocytosis—the clearance of apoptotic cells—limits inflammation and supports repair [51]; it can skew macrophages toward reparative phenotypes under appropriate stimuli [52]. We hypothesize that IR-induced DNA damage and apoptosis enhance efferocytosis and favor M2-like polarization. In this context, Mg^2+^ may further reinforce a reparative program by dampening NF-κB activity, limiting ROS, and sustaining HIF-1α/VEGFA signaling, thereby providing secreted factor–mediated support to HUVECs.

Restoring microvascular function is key to breaking the “hypoxia–inflammation–bone resorption” cycle. The microvasculature is an early target in RIBI, particularly H-type vessels (CD31^+^/Emcn^+^), leading to reduced perfusion and chronic hypoxia, which secondarily amplify oxidative stress and inflammation and drive persistent tissue injury [4, 53]. It has been reported that transient increases in local blood flow within 3 days post-IR, suggesting an intrinsic but ultimately insufficient compensatory response to augment perfusion and oxygen delivery [54]. In other progressive injury conditions, improving angiogenesis and blood flow can lessen hypoxia, genomic instability, and DNA damage [55].

In our study, under macrophage-conditioned cues, 10–20 mM Mg^2+^ significantly improved post-IR endothelial survival, migration, and tube formation. *In vivo*, beyond increasing overall microvessel density, Mg@Alg enhanced H-type vessel-related signals and increased pericyte coverage. These changes suggest not only more vessels but also better function and deeper perfusion. Improved perfusion and oxygenation may, in turn, reduce tissue stress and injury and decrease M1-like macrophage infiltration. Faster edema resolution on MRI is consistent with microcirculatory improvement, although validation with perfusion imaging and tissue oxygenation metrics is still needed.

At the bone remodeling level, IR-induced hypoxia and oxidative stress foster an inflammatory microenvironment, activating NF-κB and promoting osteoclastogenesis [15, 56]. Here, Mg^2+^ enhanced microvascular perfusion, increased the M2-like fraction to constrain inflammatory amplification; together, these effects reduced osteoclast activation. Notably, in non-surgical regions of irradiated bone, osteoclast activation was also attenuated near the marrow cavity in the Mg@Alg group, suggesting that Mg^2+^ may diffuse via the marrow and influence resorption in native bone. But spatial distribution and tissue concentration data are required to validate this.

A single high dose of radiation more readily induces acute effects, including immune dysfunction and microcirculatory abnormalities, than fractionated daily doses [34, 37]. Therefore, we used a single irradiation dose of 20 Gy, which simulates the clinical radiation dose of 50–70 Gy typically administered to head and neck cancer patients [4, 14, 57]. Given the limited influence of biomechanical loading over the short observation window, and the confounders inherent to mandibular radiation injury models—such as bacterial infection and delayed-onset osteoporotic changes [58]—we chose the rat femoral radiation model as a simplified *in vivo* platform. Although the femoral model is reproducible and histologically accessible, and repair in our study proceeded rapidly via intramembranous ossification [59], it does not recapitulate mandibular anatomy, trabecular structure, or masticatory loading. Future work will use mandibular models with clinical fractionation and load-bearing to improve translational relevance.

In this study, we optimized both the delivery form and the timing of Mg^2+^ to mitigate RIBI through iterative trials. We used a simple, well-established alginate hydrogel as the sustained-release matrix [60]. Although Mg^2+^ is not a classic alginate gelling ion, building on Topuz *et al*. [61] and our own tests, we confirmed slow-gelation crosslinking of SA by Mg^2+^. Specifically, we chemically crosslinked 5 wt% SA with 525 mM Mg^2+^ to produce Mg@Alg, which was applied locally to the rat femur to maintain therapeutic levels via sustained release and to avoid trauma from systemic administration or repeated injections—particularly important for fragile bone after IR. Aligning with clinical practice in which preventive dental procedures are scheduled 2–4 weeks before radiotherapy, and considering rodents’ 4- to 6-fold faster metabolism[54, 62], Mg@Alg was administered one week prior to IR.

Across *in vivo* and *in vitro* assays, the radioprotective effect of Mg^2+^ was time dependent, underscoring the need to target the peri-IR window. EDS indicated a reduced local Ca fraction near Mg@Alg, consistent with Mg^2+^ acting as a Ca antagonist [19]. *In vitro* sustained-release experiments confirmed that the hydrogels released Mg^2+^ completely within 2 weeks, ensuring that high concentrations of Mg^2+^ in the bone region were concentrated around the radiotherapy period. This approach avoids the risk of long-term low Ca^2+^ levels, such as abnormal osteogenesis and mineralization [18, 63], as well as prolonged HIF activation that could potentially result in tumor recurrence [40, 64] and increased risk of fibrosis [65]. Although Mg@Alg demonstrated effective localized, short-term radioprotection in this study, its release precision remains suboptimal. To further investigate the role of Mg^2+^ concentration in the molecular mechanisms of RIBI, more precise drug-delivery systems, such as microspheres, are required. Additionally, employing the second near-infrared window technology to enhance monitoring of the sustained-release range and dosage of Mg would be a promising solution [66]. Given the enhanced radioprotective effects of Mg^2+^ on bone, if Mg^2+^-based biomaterials are considered for use as dressings in non-wound areas, improvements in mechanical strength are necessary to maintain structural stability, along with considerations for drug penetration into the bone cortex.

## Conclusion

This study demonstrates that localized, short-term delivery of Mg^2+^ via a Mg^2+^-enriched alginate hydrogel to irradiated bone enhances microvascular function and promotes M2-like macrophage polarization, thereby reducing cellular injury, suppressing osteoclast activation, and limiting early bone loss and supporting bone homeostasis. Our findings also suggest that irradiation modulates Mg^2+^ mechanisms: Mg^2+^ appears to act, at least in part, through iron-dependent modulation of the PHD2/HIF-1α axis to improve immunovascular coupling; conversely, although efficacy increased under irradiation, the effective and safe concentration window narrowed. This work focuses on acute peri-radiotherapy effects (≤3 weeks) and does not evaluate long-term bone remodeling or risks related to fibrosis or tumor biology. Direct evidence is still needed for PHD2 enzymatic activity, HIF-1α proline hydroxylation, and measures of perfusion and tissue oxygenation. Future studies should incorporate fractionated irradiation and mandibular models, develop more precise local drug-delivery systems and test combinations of Mg^2+^ with other radioprotective agents to advance clinical translation for radiotherapy-associated bone preservation and repair.

## Supplementary Material

rbaf118_Supplementary_Data
