# Supplementary material for: Magnesium ion hydrogel enhances resistance to radiation-induced bone injury by modulating the bone immune microenvironment and promoting microvascularization
Source: Regen Biomater. 2025 Dec 3;12:rbaf118. doi: 10.1093/rb/rbaf118 (PMC12714384; doi:10.1093/rb/rbaf118)
Supplement: rbaf118_Supplementary_Data [file rbaf118_supplementary_data.zip › 29-Nov-2025_114426_Supplementary-data-reviesd.docx]

**Supplementary Data**

*Qiong Wang †, Xinpeng Hu* *†, Zeyu Xiao †, Kunlin Ye , Jia Li , Jiaxin Tan , Nuonuo Rao , Dong Zhang , Guodong Sun, Mingxiang Cai , Ni Shao , Nianlan Cheng , Le Bai , Xiangning Liu *, Changzheng Shi*, Liangping Luo**

**Table S1**. Primers used in q-PCR.

| Target gene | Forward primer (5′-3′) | Reverse primer (5′-3′) |
| --- | --- | --- |
| *GAPDH* | CCTCGTCCCGTAGACAAAATG | TGAGGTCAATGAAGGGGTCGT |
| *HIF-1α* | TTGCTTTGATGTGGATAGCGATA | CATACTTGGAGGGCTTGGAGAAT |
| *HIF-2α* | GACAAGACCATCAGTGCGAACAT | CTGGAACTGGGAGGCATAGC |
| *BMP-2* | GCTGACCACCTGAACTCCACT | CCTCCACAACCATGTCCTGATAA |
| *TNF-α* | ACCCTCACACTCACAAACCA | ATAGCAAATCGGCTGACGGT |
| *IL-10* | AATAAGCTCCAAGACCAAGGTGT | CATCATGTATGCTTCTATGCAGTTG |
| *CCl-5* | TGCTCCAATCTTGCAGTCGT | GCAAGCAATGACAGGGAAGC |
| *VEGF* | AGGAGTACCCCGACGAGATAGA | CACATCTGCTGTGCTGTAGGAA |
| *OSM* | AATCGTGGCTGCTCCAACTCT | TTCAGGTTTTGGAGGCGGATA |

**
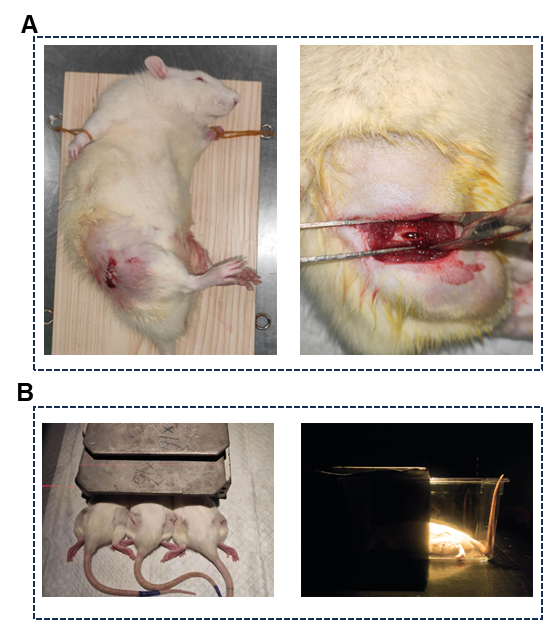
**

**Figure S1.** Surgical procedure in rat model. (A) Images taken during the animal surgery. (B) Portions of the rats were irradiated 1 week post-operation.

**
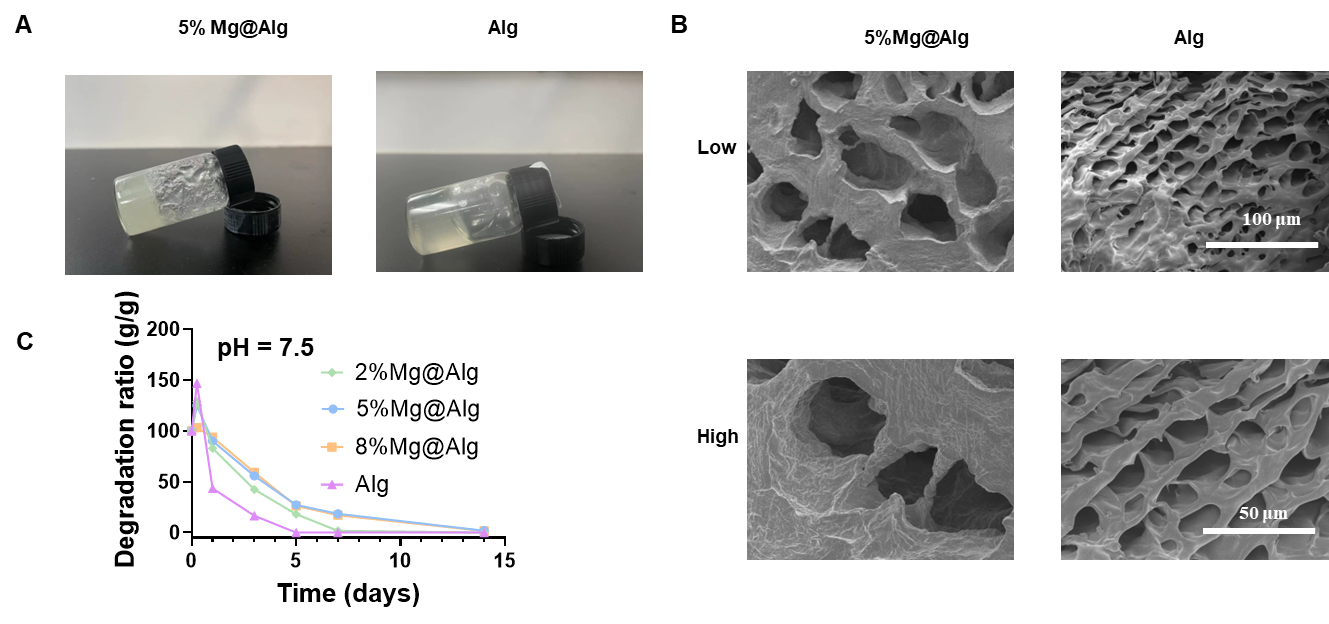
Figure S2.** Morphological characteristics of hydrogels. (A) Images of hydrogels. (B) Representative SEM images of hydrogels in the dry state. (C) Degradation behavior of hydrogels at pH =7.5 (*n* = 3).


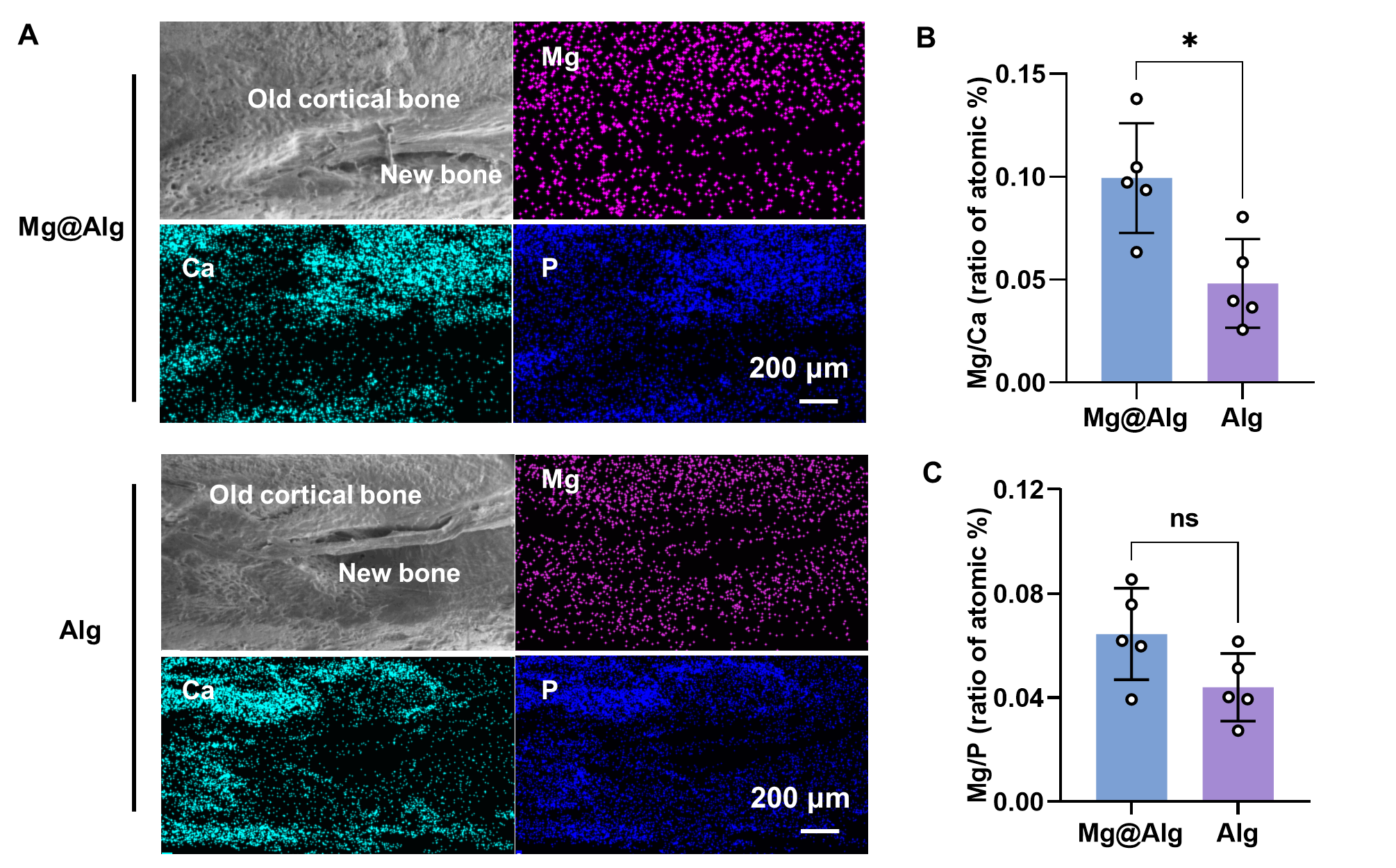


**Figure S3.** Representative SEM-EDS images and atomic ratios of Mg@Alg and Alg group in and around the defects at 7 days post-operation. (A) Representative SEM images and EDS mapping of Mg, P and Ca in both the new bone and the surrounding old cortical bone. Quantitative analysis of the (B) Mg/Ca and (C) Mg/P atomic ratios in the new bone areas (*n* = 5). Data are mean ± SD. ns *P*＞ 0.05, * *P* < 0.05.


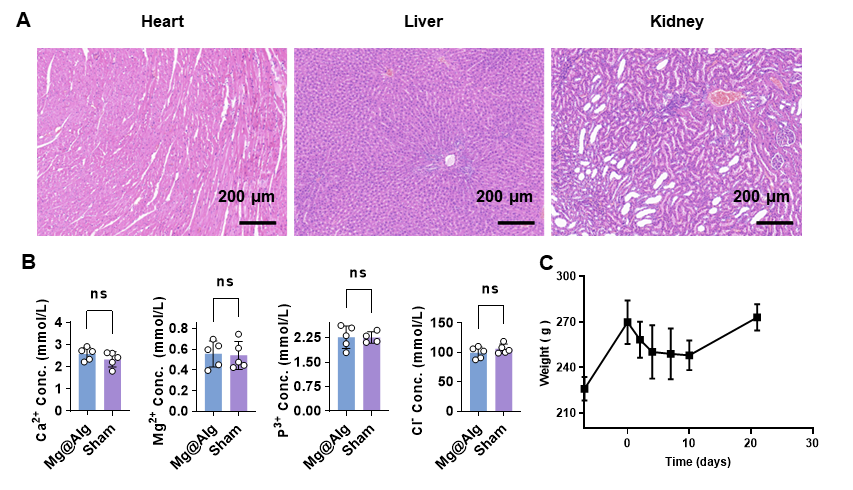


**Figure S4.** Systemic biocompatibility responses of the hydrogels. (A) Histological morphology of rat heart, liver, and kidney tissues at 14 days post-operation. (B) Serum concentrations of Mg, Ca, P, and Cl ions in rats at 14 days post-operation, compared with the sham-operated group (*n* = 5). (C) Weight changes in rats post-operation, with the time point of IR (7 days post-operation) defined as the baseline (*n* = 7). Data are mean ± SD. ns *P* ＞ 0.05.


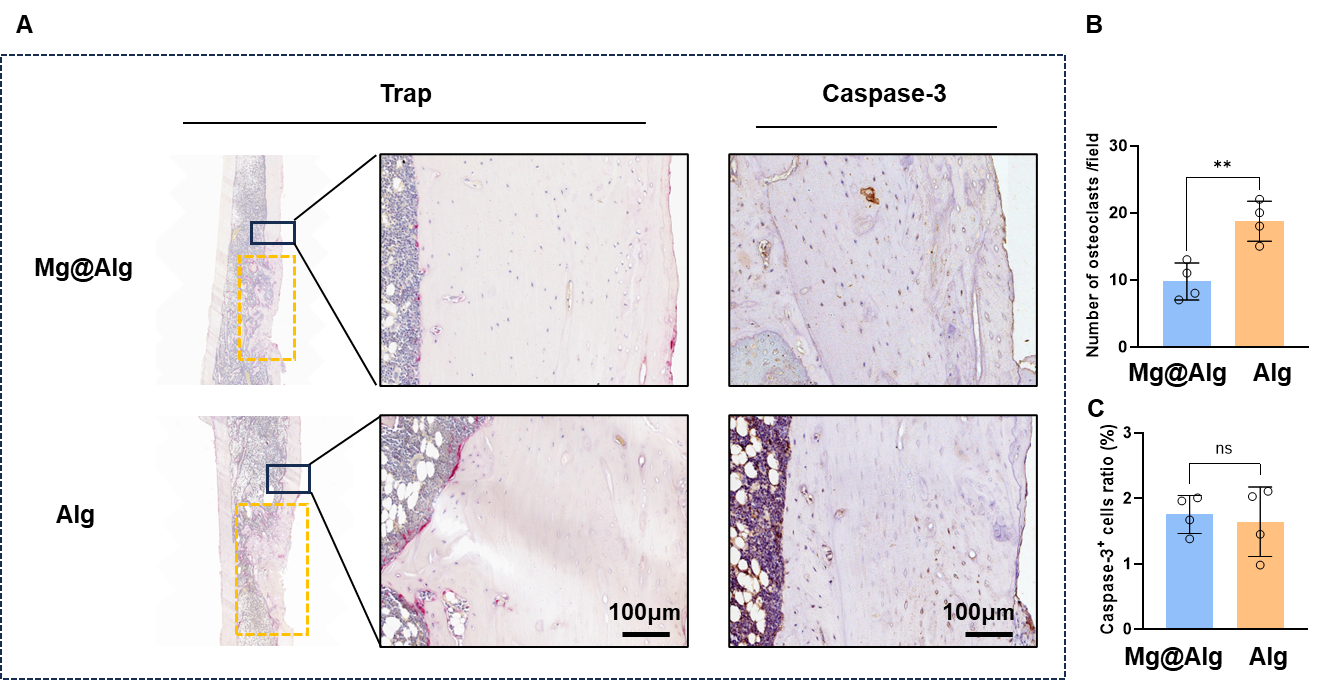


**Figure S5.** Representative data of Trap^+^ osteoclasts and Caspase-3^+^ in the non-surgical region of the irradiated bone at 14 days post-IR. (A) Representative images of Trap^+^ osteoclasts and immunohistochemical images of Caspase-3. Quantitative analysis of (B) Trap^+^ and (C) Caspase-3^+^ (*n* = 4). Data are expressed as mean ± SD. n.s. *P* > 0.05. ***P* < 0.01.


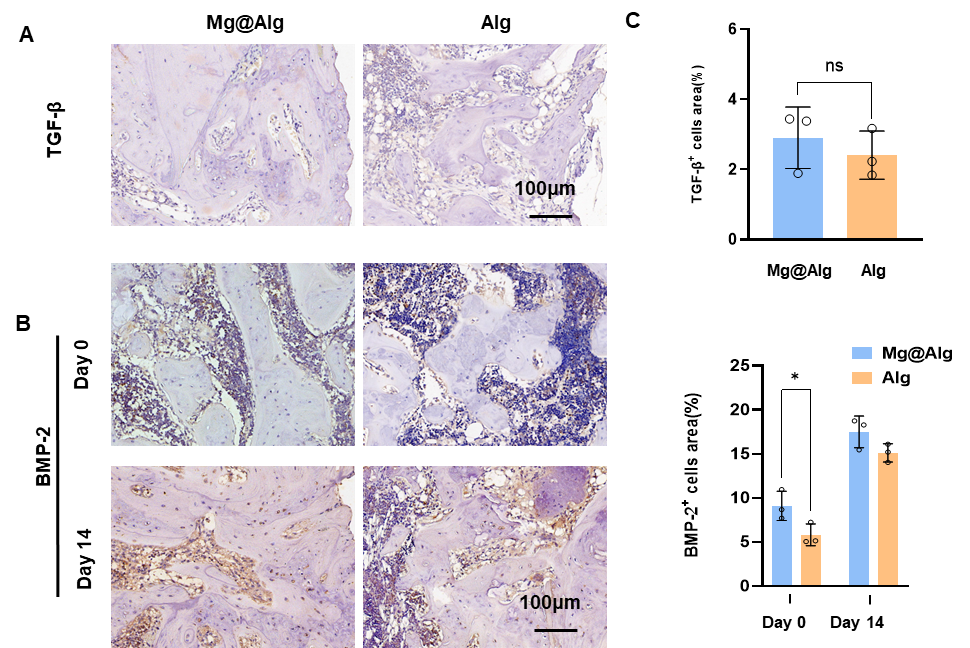


**Figure S6.** Representative immunohistochemical images and quantitative analysis of BMP-2 and TGF-β in defects post-IR. Representative immunohistochemical images of (A) TGF-β and (B) BMP-2. (C) Quantitative analysis of TGF-β^+^ and BMP-2^+^ (*n* = 3). Data are expressed as mean ± SD. n.s. *P* > 0.05.


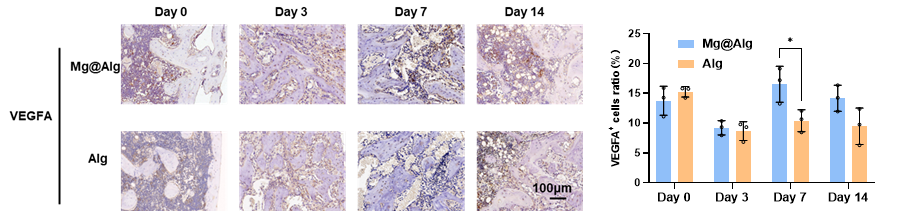


**Figure S7.** Representative immunohistochemical images and quantitative analysis of VEGFA in defects at 0,3,7 and 14 days post-IR (*n* = 3). Data are mean ± SD. * *P* < 0.05. No significant differences between the other groups.


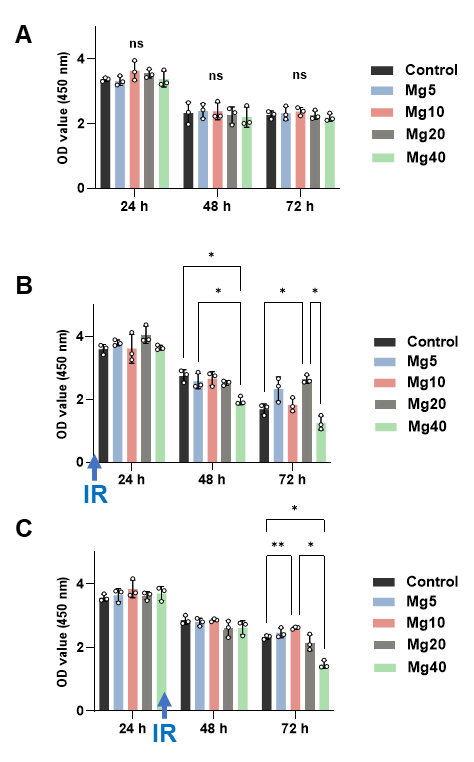


**Figure S8.** Effect of Mg²⁺ concentrations on cell proliferation under various conditions within 72 h (*n* = 3). (A) Under normal conditions. (B) Mg^2+^ addition immediately after IR. (C) 24-hour Mg^2+^ pre-treatment followed by IR. All the time points were calculated from the first addition of Mg^2+^. Data are presented as mean ± SD. **P* < 0.05, ***P* < 0.01. No significant differences between the other groups.


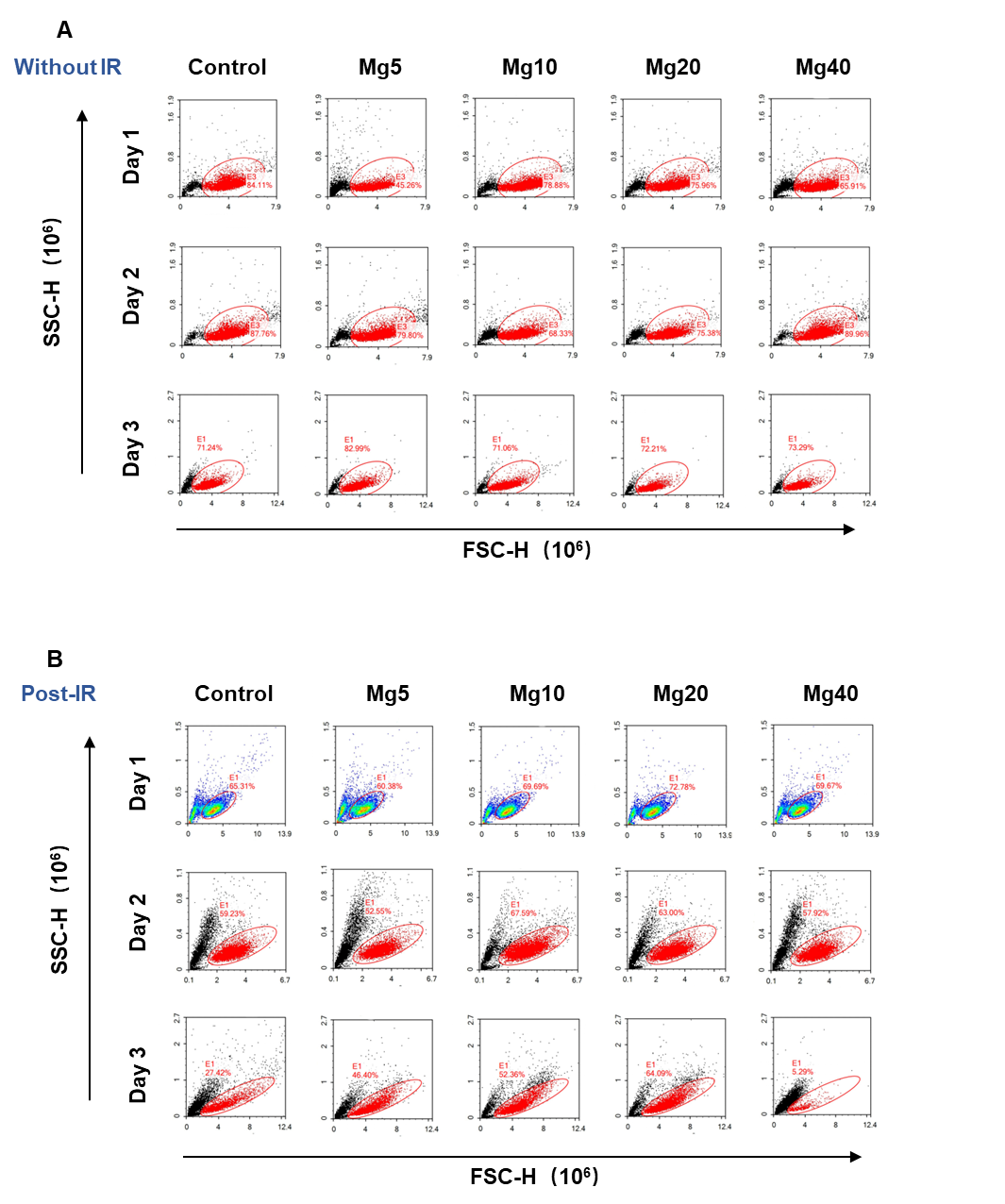


**Figure S9.** Gate cells based on forward scatter (FSC) and side scatter (SSC) before staining markers in flow cytometry experiments. Gate information of cells (A) before and (B) after IR. IR induces significant cell death at 72 h in the control group, while Mg^2+^ partially alleviates this effect.


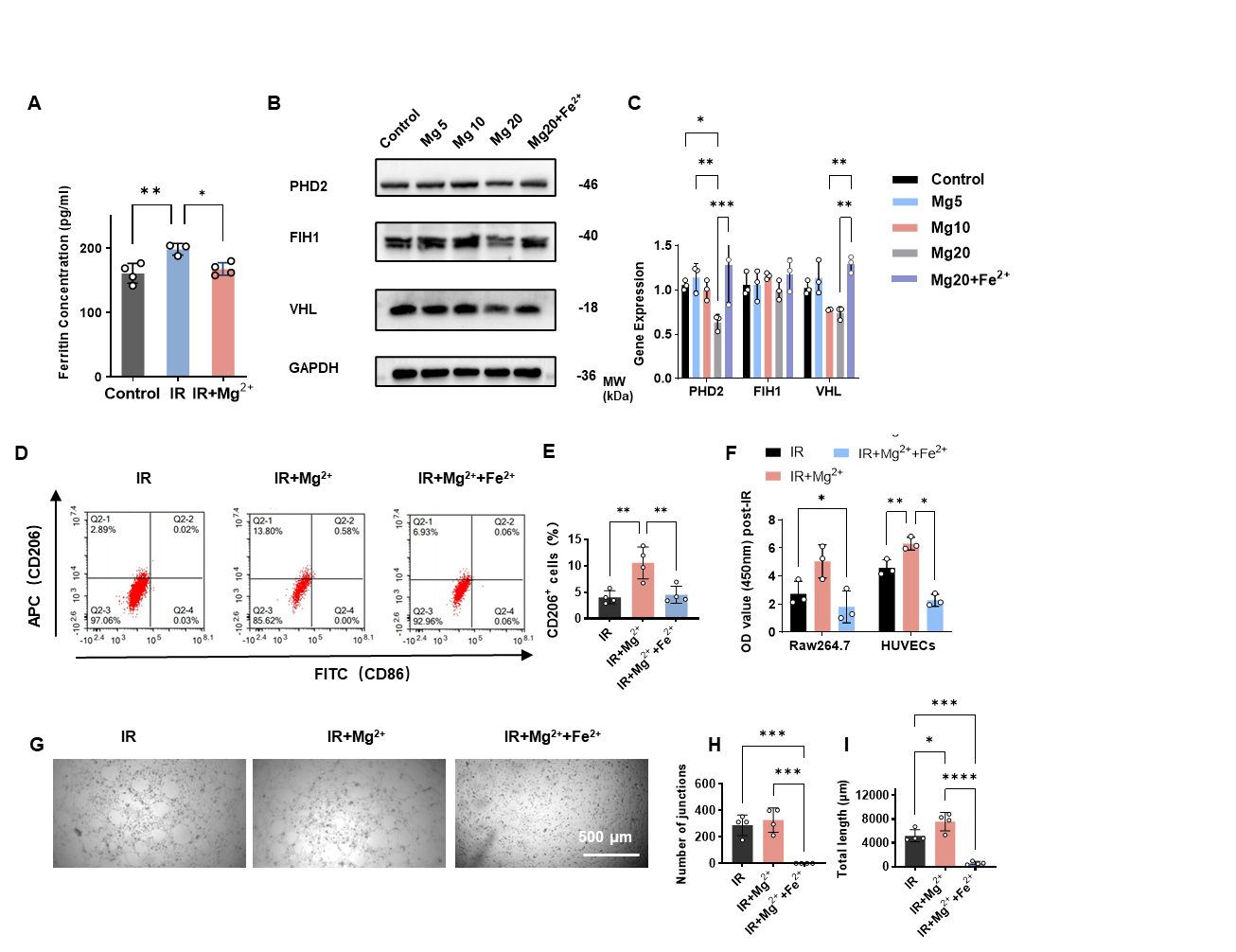


**Figure S10**. Effect of Mg²⁺ on HIF-related protein expressions and Fe^2+^ supplementation experiments. (A) Ferritin levels in RAW 264.7 cells (*n* = 4). (B) Western blot and (C) q-PCR analysis of PHD2, FIH1 and VHL expression after exposure to a concentration gradient of Mg^2+^ and Fe^2+^ (*n* = 3). (D) Representative flow cytometry plots showing the effect of Fe^2+^ supplementation on Mg^2+^-induced M2-like polarization. (E) Percentage of CD206^+^ cells (*n* = 4). (F) Effects of Mg²⁺ and Fe²⁺ treatment on the cell proliferation under IR condition (*n* = 3). (G) Representative images of HUVEC tube-formation assays with Mg^2+^ and Fe^2+^ treatments. Quantifications of (H) the number of junctions and (I) total length in tube formation (*n* = 4). Data are expressed as mean ± SD. * *P* < 0.05, ** *P* < 0.01. ****P* < 0.001, *****P* < 0.0001. No significant differences between the other groups.
